# PINK1 restrains periodontitis-induced bone loss by preventing osteoclast mitophagy impairment

**DOI:** 10.1016/j.redox.2023.103023

**Published:** 2023-12-30

**Authors:** Ji Sun Jang, Seo Jin Hong, Shenzheng Mo, Min Kyung Kim, Yong-Gun Kim, Youngkyun Lee, Hong-Hee Kim

**Affiliations:** a Department of Cell and Developmental Biology, Dental Research Institute, School of Dentistry, Seoul National University, Seoul, 03080, Republic of Korea; b Department of Periodontology, School of Dentistry, Kyungpook National University, Daegu, 41940, Republic of Korea; c Department of Biochemistry, School of Dentistry, Kyungpook National University, Daegu, 41940, Republic of Korea

**Keywords:** PINK1, Osteoclast, Mitophagy, ROS, Periodontitis

## Abstract

The oral colonization of periodontal pathogens onto gingival tissues establishes hypoxic microenvironment, often disrupting periodontal homeostasis in conjunction with oxidative stress. The association between reactive oxygen species (ROS) and osteolytic periodontitis have been suggested by recent studies. PTEN-induced kinase 1 (PINK1), a mitochondrial serine/threonine kinase, is an essential protein for mitochondrial quality control as it protects cells from oxidative stress by promoting degradation of damaged mitochondria through mitophagy. However, the pathophysiological roles of PINK1 in osteoclast-mediated bone loss have not been explored. Here we aimed to determine whether PINK1 plays a role in the regulation of osteoclastogenesis and alveolar bone resorption associated with periodontitis. C57BL/6 wild type (WT) and *Pink1* knockout (KO) mice were subjected to ligature-induced periodontitis (LIP), and alveolar bones were evaluated by μCT-analysis and tartrate-resistant acid phosphatase (TRAP) staining. The μCT-analysis showed that bone volume fraction and travecular thickness were lower in *Pink1* KO compared to WT mice. The number of TRAP-positive osteoclasts was markedly increased in the periodontal tissues of *Pink1* KO mice with LIP. The genetic silencing or deletion of *Pink1* promoted excessive osteoclast differentiation and bone resorption *in vitro*, as respectively indicated by TRAP staining and resorption pits on dentin slices. PINK1 deficiency led to mitochondrial instabilities as indicated by confocal microscopy of mitochondrial ROS, mitochondrial oxygen consumption rate (OCR) analysis, and transmission electron microscopy (TEM). Consequently, a significant increase in Ca^2+^-nuclear factor of activated T cells 1 (NFATc1) signaling was also found. On the other hand, restoration of mitophagy and autophagy by spermidine (SPD) treatment and the resolution of oxidative stress by *N*-acetyl-l-cysteine (NAC) treatment protected PINK1 deficiency-induced excessive generation of osteoclasts. Taken together, our findings demonstrate that PINK1 is essential for maintaining mitochondrial homeostasis during osteoclast differentiation. Therefore, targeting PINK1 may provide a novel therapeutic strategy for severe periodontitis with fulminant osteolysis.

## 1 Introduction

Periodontitis, a chronic inflammatory disease that accompanies an irreversible osteolysis of alveolar bone, is caused by an excessive activation of host immune responses against continuously accumulating oral pathogens in the gingival tissue [1]. Recently, the importance of regulating mitochondrial abnormalities and production of ROS has been emerged in the pathogenesis of periodontitis [2]. For instance, oxidant levels were higher in serum and gingival crevicular fluid of chronic periodontitis patients [3]. The ROS production can be upregulated in human leukocytes by pathogenic bacteria or by virulent factors in periodontitis [4,5]. In addition, mitochondrial dysfunction and ROS production were detected in human gingival fibroblasts from periodontitis patients [6]. Although previous studies have emphasized that mitochondrial stress and ROS production are detrimental to periodontal health, the importance of maintaining mitochondrial homeostasis in osteoclasts during periodontitis has not been clearly elucidated.

Osteoclasts, myeloid lineage-originated and multinucleated bone-resorbing cells, accumulate a vast number of mitochondria through multiple cellular fusion events and mitochondrial biogenesis to fulfill their energy requirements [7]. Recently, mitochondrial stress has been implicated in excessive osteoclast differentiation. Defects in the mitochondrial electron transport chain activates Ca^2+^/calcineurin-NFATc1 pathway and subsequent osteoclast differentiation due to a burst release of mitochondrial Ca^2+^ ion to the cytosol [8]. Similarly, bone marrow macrophages (BMMs) from mitochondrial inner membrane protein MPV17 deficient mice, a model for mitochondrial DNA depletion, were more prone to differentiate into osteoclasts [8]. Mitochondrial homeostasis is maintained through fusion, fission, biogenesis, and mitophagy [9], and each of these aspects except mitophagy has recently been noticed in osteoclasts. Previously, we reported that mitofusin 2 may maintain mitochondrial fusion and Ca^2+^/calcineurin-NFATc1 axis for proper osteoclast differentiation [10]. In addition, genetic depletion of dynamin-related protein 1, a mitochondrial fission protein, was reported to suppress osteoclast formation [11]. Myeloid lineage-specific ablation of PPARγ coactivator 1 beta, an essential regulator of mitochondrial biogenesis, impaired resorptive function of osteoclasts [12]. Whereas mitochondrial biogenesis, fusion, and fission were shown to be essential for osteoclast activation and formation, the role of mitophagy in osteoclast differentiation has not been investigated.

PINK1 is a mitochondrial serine/threonine kinase that protects cells from damaged mitochondria-induced oxidative stress by promoting microtubule-associated protein 1A/1B-light chain 3 (LC3)-mediated mitophagy [13]. PINK1 is suggested to be essential for the regulation of intracellular ROS in consideration of loss of PINK1 resulting in massive mitochondrial ROS production [[14], [15], [16]]. In addition, PINK1 is involved in mitochondrial and intracellular Ca^2+^ homeostasis. Heeman and colleagues reported that knockdown of *Pink1* in mouse neuroblastoma cells and primary mouse embryonic fibroblasts impaired mitochondrial Ca^2+^ influx but increased cytosolic Ca^2+^ extrusion [17]. Besides, PINK1 is also involved in ATP synthesis. As a mitochondrial serine/threonine kinase, it induces phosphorylation of an accessory subunit of complex I, NADH:ubiquinone oxidoreductase subunit A10 protein [18]. Even more, the deletion of *Pink1* in primary mouse embryonic fibroblasts increased glucose uptake and release of lactate [19]. As PINK1 has multiple roles in cellular processes including mitochondrial quality control, ROS production, Ca^2+^ homeostasis and ATP synthesis, which are indispensable for osteoclast differentiation, we hypothesized that PINK1 may have crucial functions in proper osteoclast formation.

In this study, we investigated the function of PINK1 in osteoclast differentiation and periodontitis by using *Pink1* knock-out (KO) mice. *Pink1* KO mice developed severe periodontitis upon ligature placement. BMMs from *Pink1* KO mice were more prone to differentiation into osteoclasts with higher bone resorptive function. The loss of PINK1 led to mitochondrial dysfunction accompanied by impaired mitochondrial respiration, increased intracellular Ca^2+^ and lactate levels, excessive nuclear translocation of NFATc1, and enhanced ROS production during osteoclast differentiation. Scavenging of ROS with NAC and stimulating mitophagy and autophagy by SPD were able to suppress excessive osteoclast formation in PINK1-deficient osteoclast precursors. Here, we delineated the mechanism by which PINK1 regulates osteoclast formation and bone resorption and we suggest that maintaining proper mitochondrial homeostasis in osteoclasts can be considered as a therapeutic strategy for periodontal diseases.

## 2 Materials and methods

### 2.1 Experimental animals

All animal experiments were approved by the Institutional Animal Care and Use Committee at Seoul National University (SNU-211130-1-1), and conformed to the ARRIVE 2.0 guidelines. *Pink1 KO* mice with C57BL/6 background were obtained from Professor DW Kim at Chungnam National University College of Medicine [20]. Mice were bred in a SPF facility under 12 h shifts of light-dark cycle and free access to food and water.

### 2.2 Ligature-induced periodontitis (LIP)

The ligature method was conducted on male 11-week-old *Pink1* WT and *Pink1* KO littermates to induce periodontitis. Maxillary second molar on the left-side was ligated with 5-0 silk and knots were placed on lingual side between the first and second molars. The contralateral side was left without ligature as a control. The ligature was placed for five days.

### 2.3 μCT analysis

Mice were anesthetized with Zoletil-Rompun mixture then euthanized. Samples of maxillary alveolar bones of *Pink1* WT (*n* = 10) and *Pink1* KO (*n* = 7) littermates were collected. Alveolar bone loss of interradicular and interdental alveolar bone of maxillary second molars was assessed by μCT. Trabecular parameters of alveolar bone samples were analyzed by using SkyScan 1273 μCT scanner (70 kV, 114 μA, 9 pixel size; Skyscan, Aartselaar, Belgium). Alveolar bones were centered on the circular region-of-interest with diameter of 100-pixel size (0.9 mm) and bone parameters were analyzed for 50 frames (0.440 mm) from crown-side, 0.1 mm below of cemento-enamel junction where alveolar bone crest of *Pink1* WT controls were found on average, to root-side by using μCT-analysis software (CtAN; Skyscan) at threshold range between 90 and 255.

### 2.4 Histomorphometry

For histological study of alveolar bone osteoclasts, maxillary bones were fixed with 4 % paraformaldehyde (PFA), decalcified with 12%-EDTA solution for 4 weeks, and embedded into paraffin wax. Paraffinized tissues were sectioned with 6 μm-thickness and tissue sections were subjected to TRAP staining. Alveolar bone osteoclasts in interradicular septum area of maxillary second molar were analyzed by using the Osteomeasure software (Osteometrics, CA, USA).

### 2.5 BMMs and osteoclasts cultures

Bone marrow cells were isolated from tibiae and femurs of 5-week-old *Pink1* WT and *Pink1* KO mice and cultured overnight in α-MEM (Welgene, Daegu, Korea) containing 1 % penicillin and streptomycin and 10 % fetal bovine serum (hereafter referred to as α-MEM complete medium). BMMs were obtained by culturing non-adherent cells from the bone marrow cell culture with 30 ng/ml of macrophage colony stimulating factor (M-CSF) for 3 days. To obtain pre-osteoclasts (pOCs) and mature osteoclasts, BMMs were cultured, respectively, for 2 and 4 days in α-MEM complete medium with 100 ng/ml of receptor activator of nuclear factor-κB ligand (RANKL) and 30 ng/ml of M-CSF. In cell treatment experiments, BMMs seeded on 48-well plates were cultured with spermidine (5 μM) or NAC (2.5 mM) in the presence of RANKL and M-CSF for 3 days.

### 2.6 TRAP staining

Cultured cells were fixed with 4 % PFA for 15 min and permeabilized with 0.1 % Triton X-100. TRAP-staining of cultured cells was carried out using leukocyte acid phosphatase kit (Sigma-Aldrich, MO, USA) following the manufacturer's instructions. Osteoclast formation was assessed with a light-microscope and TRAP^+^ cells containing more than three nuclei were counted as osteoclasts.

### 2.7 Real-time PCR (RT-PCR)

Total RNA was extracted by using TRIzol (Invitrogen, CA, USA). Superscript II reverse transcriptase (Invitrogen) was used for cDNA synthesis. cDNA was amplified by using SYBR Green Master Mix reagents (Kapa Biosystems, MA, USA). Real-time PCR was carried out by using an ABI 7500 instrument (Applied Biosystems, CA, USA). The mRNA expressions were normalized to hypoxanthine phosphoribosyl transferase 1 (HPRT), and 2-ΔΔCT values were presented as fold-degree. Primer sequences are listed in Appendix Table S1.

### 2.8 Bone resorption assay

BMMs were cultured on dentin slices (Immunodiagnostic Systems, Boldon, United Kingdom) in α-MEM complete medium with 30 ng/ml of M-CSF and 100 ng/ml of RANKL for 12 days. Resorption area and pit depths of dentin slices were imaged and analyzed under a Zeiss LSM 800 laser-scanning microscope (Carl Zeiss, Oberkochen, Germany).

### 2.9 Cytoplasmic Ca^2+^ measurement

pOCs were incubated with 5 μM Fluo-4-dye-loading solution in Hanks' balanced salt solution (HBSS) at 37 °C for 30 min according to the manufacturer's instructions. Cells were then washed and resuspended in HBSS. The fluorescence intensity of Fluo-4 Ca^2+^ indicator was measured at excitation 488 nm and emission 505–530 nm.

### 2.10 Western blot

Cells were lysed in RIPA buffer containing protease inhibitor cocktail (Sigma-Aldrich, MO, USA). Protein concentration was determined by using Pierce BCA protein assay kit (Thermo Scientific, MA, USA) with bovine serum albumin for standardization. Equal amounts of total cell lysates were analyzed by SDS/PAGE with a 10 % or 15 % separation gel. Band density was measured by using the ImageJ program.

### 2.11 Immunocytochemistry of nuclear NFATc1

To detect nuclear translocation of NFATc1 in pOCs, BMMs were cultured for 2 days in the presence of RANKL and M-CSF on cover-glasses. Serum starvation was applied for 4 h and cells were re-stimulated with RANKL (500 ng/ml) for 15 min. Cells were fixed with 3.7 % formaldehyde and permeabilized with 0.1 % Triton X-100 for 30 min at room temperature. Cells were then blocked with 1 % bovine serum albumin and incubated with *anti*-NFATc1 antibody (Santacruz, TX, USA) (1:200) at 4 °C overnight. Cells were washed and incubated with fluorochrome-conjugated secondary antibody (1:200) (Thermo Fisher Scientific, MA, USA) for 1 h. For nuclear staining, 4′,6-diamidino-2-phenylindole (DAPI) was used. Fluorescence signals were detected under a LSM700 microscope (Carl Zeiss).

### 2.12 Bioinformatic analysis (RNA sequencing, GO analysis)

BMMs of each group, *Pink1* WT and *Pink1* KO, were cultured in α-MEM complete meium with M-CSF (30 ng/ml) and RANKL (100 ng/ml) for 1 day. RNAs of each sample from two of biological replicates were prepared and bulk RNA sequencing was performed by Ebiogen (Seoul, Korea). The data of GSE57468 was obtained from Gene Expression Omnibus database (http://www.ncbi.nlm.nih.gov/geo/). Gene ontology (GO) enrichment analysis of differentially expressed genes (DEGs) with p-value <0.05 was performed by using Database for Annotation, Visualization and Integrated Discovery (DAVID) (https://david.ncifcrf.gov/, 6.7 version).

### 2.13 Transmission electron microscopy (TEM)

Cells were fixed with Karnovsky's fixative (2 % glutaraldehyde, 2 % PFA, 0.5 % CaCl_2_) solutions, dehydrated, and then embedded in resin. Ultrathin sections were placed on a copper grid and stained with uranyl acetate and lead citrate. Mitochondria were imaged using JEM-1011 TEM (JEOL, Tokyo, Japan) at 80 kV.

### 2.14 Measurement of mitochondrial membrane potential (ΔΨm)

ΔΨm was measured by using the 5,5′, 6,6′-tetrachloro-1,1′, 3,3′-tetraethyl benzimidazolyl carbocyanine iodide (JC-1) mitochondrial membrane potential detection kit (Biotium, CA, USA) according to the manufacturer's instruction. The fluorescence was detected with a confocal laser scanning microscope (Carl Zeiss) under excitation at 590 nm and emission at 610 nm for red fluorescence and excitation at 490 nm and emission at 520 nm for green fluorescence.

### 2.15 Measurement of intracellular and mitochondrial ROS

Intracellular and mitochondrial ROS levels were measured by using 10 μM of 2,7-dichlorofluorescein diacetate (CM-H_2_DCFDA) and 5 μM of MitoSOX™ (Invitrogen), respectively, under serum-free condition at 37 °C for 15 min.

### 2.16 Seahorse XFe96 analysis

Cellular oxygen consumption rate (OCR) was monitored using the Seahorse XFe96 system (Agilent Technologies, CA, USA). *Pink1 WT* and *Pink1 KO* pOCs (100,000 cells/well) were seeded on a XF96 cell culture microplate. An XFe96 cartridge was hydrated and incubated overnight in a non-CO_2_ incubator at 37 °C. On the day of run, d-glucose (1.25 ml), pyruvate (630 μl) and glutamate (1 ml) were added to the 50 ml Seahorse analysis medium and placed in a 37 °C incubator prior to use. The XFe96 cell culture microplate was washed and the final volume of 180 μl of Seahorse assay medium was added to the cell. The means to measure respiration were loaded onto the drug ports of a hydrated sensor cartridge in the following order: oligomycin (2 μM), carbonyl cyanide-*p*-trifuloromethoxyphenylhydrazon (FCCP; 1 μM), and antimycin A + rotenone (0.5 μM). In analyses of extracellular acidification rate (ECAR), glucose (10 mM), oligomycin (1 μM), and 2-DG (50 mM) were sequentially added. Prior to the analysis, for normalization and cell-counts, the XFe96 cell culture microplate was placed in Cytation-1 imaging multi-mode reader (BioTek, VT, USA). Data were analyzed using the Wave 2.6.1 Software (Agilent Technologies, CA, USA).

### 2.17 Lactate measurement

3 × 10^5^ of BMMs from *Pink1* WT and *Pink1* KO were seeded in 6-well plates and cultured with osteoclastogenic medium for 2 days to form pOCs. Intracellular lactate levels of each group were measured by using the Colorimetric/Fluorometric assay Kit (Biovision, Waltham, MA, USA) according to manufacturer's instructions.

### 2.18 ATP measurement

3 × 10^3^ of BMMs from *Pink1* WT and *Pink1* KO were seeded on 96-well plates and cultured with osteoclastogenic medium for 2 days. Intracellular ATP levels were measured with a luminescent ATP detection assay kit (Abcam, Cambridge, MA, USA) in accordance with manufacturer's guidelines.

### 2.19 Mitophagy measurement

Cells on glass-coverslips were incubated with 0.5 nM of MitoTracker Red CMXRos probe (Invitrogen, CA, USA) for 45 min according to the manufacturer's instruction. Cells were fixed with 4 % PFA for 15 min at room temperature and permeabilized with 0.1 % Triton X-100. Cells were blocked in 1 % bovine serum albumin for 2 h and incubated with LC3 primary antibody (Cell Signaling Technology, MA, USA) overnight at 4 °C. Primary antibodies and nuclei were labeled with Alexa488-conjugated anti-rabbit secondary antibodies (Jackson Immuno Research Laboratories, PA, USA) and DAPI, respectively. The fluorescence was detected by using an LSM700 or LSM980 confocal microscope (Carl Zeiss). To measure mitophagy, the mitochondrial LC3 puncta was determined by using the ImageJ software. The mitochondrial region of interest (ROI) was set by ImageJ with a threshold of red-fluorescence intensity (MitoTracker). ROIs for LC3 puncta within mitochondria were then set with respective threshold of fluorescence intensity of merged images of red channel (MitoTracker) and green channel (LC3). Thresholds for merged channels were validated by JACoP plugin in the ImageJ software. The number of mitochondrial LC3 puncta was counted and mean fluorescence intensity (MFI) of LC3 on mitochondria was measured.

### 2.20 Autophagy flux analysis

BMMs from *Pink1 WT* and *Pink1 KO* seeded in 24-well plates with cover-glasses were transfected with mRFP-GFP-LC3 (plasmid # 84572; Addgene, MA, USA) using polyfect reagent (Promega, WI, USA) for 4.5 h. Transfected BMMs further cultured in the presence of RANKL and M-CSF for 2 days. After fixing with 3.7 % PFA, images of cells were obtained using an LSM700 confocal microscope (Carl Zeiss). For quantification of autophagic flux, GFP^−^/RFP^+^ (red) puncta or GFP^+^/RFP^+^ (yellow) puncta were counted.

### 2.21 Statistical analysis

The statistical significance between two groups was assessed by student's *t*-test. *P* values less than 0.05 were considered to be significant. All data were presented as the means with standard deviations.

## 3 Results

### 3.1 LIP-induced alveolar bone loss is greater in Pink1 KO mice compared to Pink WT mice

**Fig. 1:**
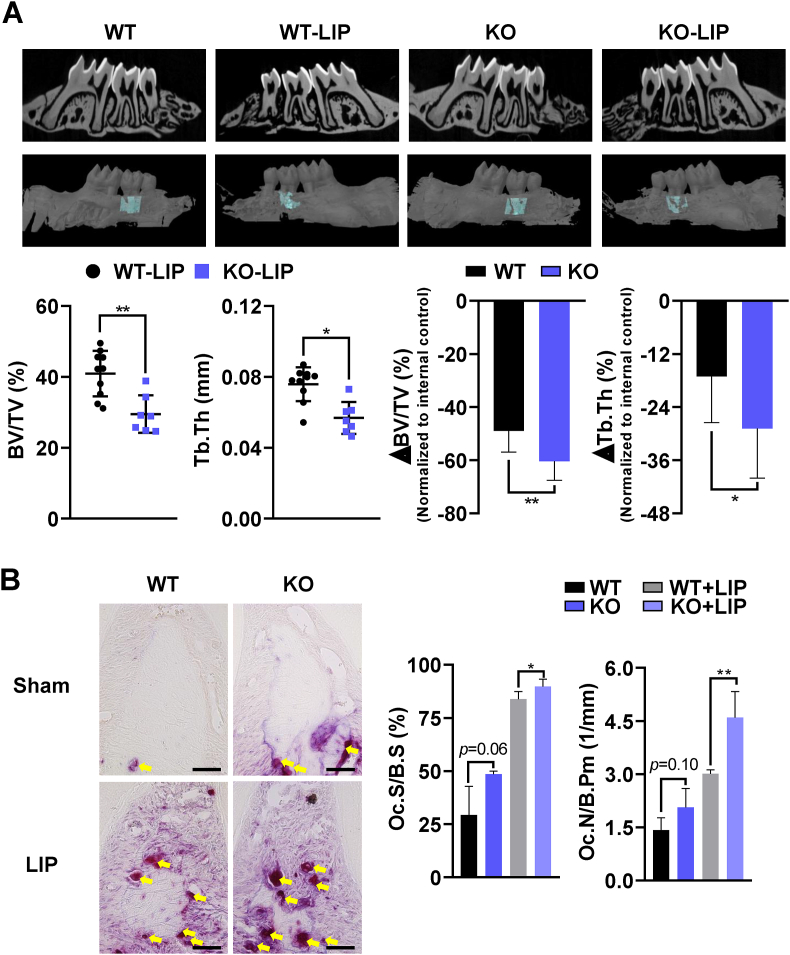
Genetic deletion of *Pink1* exacerbates ligature-induced periodontitis. (A) Two- and three-dimensional μCT images of sham and silk-ligated *Pink1* WT (*n* = 10) and *Pink1* KO (*n* = 7) littermates. The bone parameters of alveolar bone region of maxillary second molars, indicated by turquoise color, were assessed by μCT-analysis (dot graphs). The relative reductions of bone parameters of ligated sides compared to the contralateral un-ligated sides are shown as bar graphs (B) TRAP staining images of periodontal tissues of *Pink1* WT and *Pink1* KO littermates (at 400× magnification). TRAP^+^ cells were indicated by yellow arrows. Scale bar, 50 μm. TRAP^+^ cells on alveolar bone surfaces were assessed. The osteoclast surfaces and numbers were measured at 200× magnification with the Osteomeasure software. Oc.N/B.*Pm*, Osteoclast number/bone perimeter, Oc.S/B.S, Osteoclast surface/bone surface. *P < 0.05 and **P < 0.01. (For interpretation of the references to color in this figure legend, the reader is referred to the Web version of this article.)

Previous studies have suggested that mitochondrial dysregulation and ROS production are linked to pathology of periodontitis [3,4,6]. Given that shoulders multiple mitochondrial activities including mitophagy, ATP synthesis, ROS production, and Ca^2+^ release, PINK1 may be responsible for maintaining periodontal health. To explore the role of PINK1 in inflammatory periodontitis, silk-ligatures were placed on the second molar of 11-week-old *Pink1* WT and *Pink1* KO mice. Both *Pink1* WT-LIP and *Pink1* KO-LIP groups displayed typical features of periodontitis upon ligature placements (Fig. 1A, top panel). Results of μCT-analyses of alveolar bones showed that bone volume fraction (BV/TV) and trabecular thickness (Tb. Th) were significantly lower in *Pink1* KO-LIP than in *Pink1* WT-LIP groups (Fig. 1A, bottom left). Also, the extent of reductions in BV/TV and Tb. Th caused by LIP was greater in *Pink1* KO mice (Fig. 1A, bottom right). We next evaluated osteoclasts on interradicular alveolar bone surface by TRAP staining (Fig. 1B, left). The number of TRAP^+^ cells on the alveolar bone surfaces (Oc.N/B.pm) were increased in both *Pink1 WT*-LIP and *Pink1 KO*-LIP groups, but the increase was more prominent in *Pink1 KO* group (Fig. 1B, right). In comparison between LIP groups, the percentate of osteoclast surface per bone surface (Oc.S/B.S) as well as Oc.N/B.pm was significantly higher in *Pink1* KO than in *Pink1* WT mice (Fig. 1B, right). Together, these results indicate that PINK1 deficiency aggravates alveolar bone loss induced by experimental periodontitis in mice.

### 3.2 PINK1 deficiency enhances osteoclast formation and bone resorption, and promotes nuclear translocation of NFATc1

**Fig. 2:**
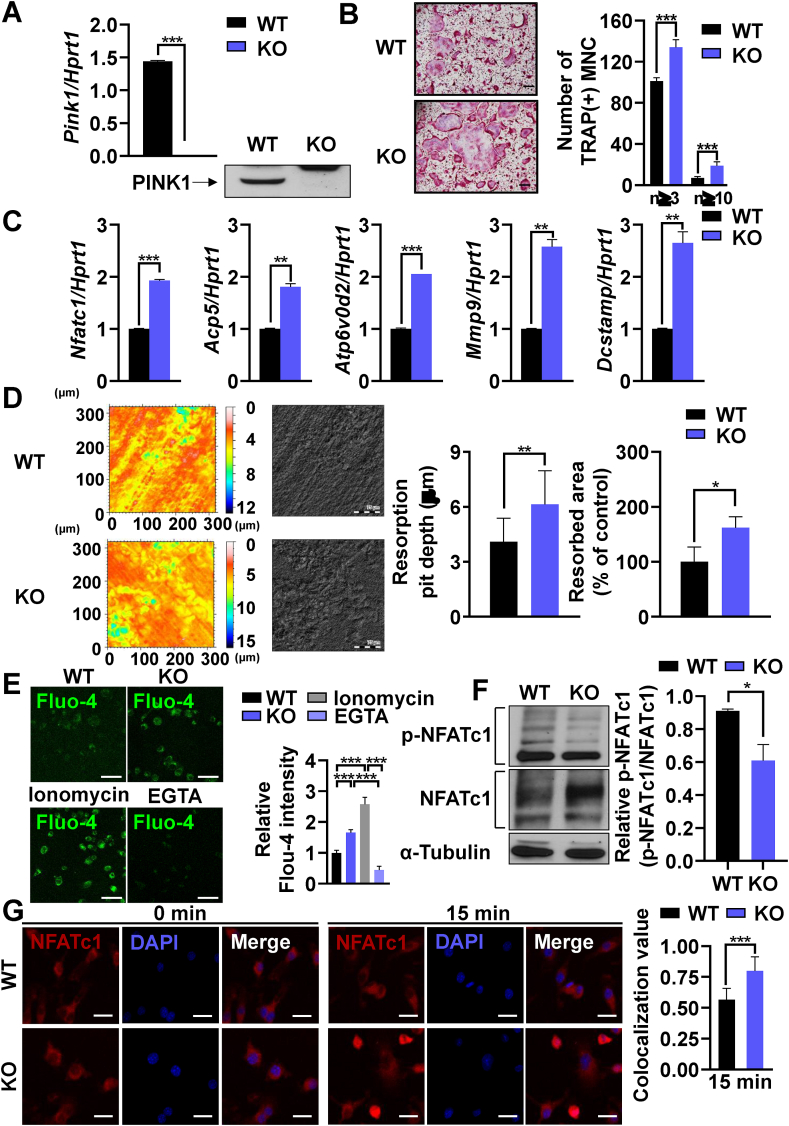
PINK1 inhibits osteoclastogenesis through the Ca^2+^-NFATc1 axis. (A) BMMs isolated from *Pink1* WT or *Pink1* KO mice were subjected to RT-PCR and western blotting. ***P < 0.001. (B) *Pink1* WT or *Pink1* KO BMMs were cultured in the presence of RANKL and M-CSF for 3 days and stained for TRAP. The number of TRAP^+^ multinucleated cells with more than 3 or 10 nuclei are presented. MNC, multinucleated cells. Scale bar, 200 μm. (C) BMMs isolated from *Pink1* WT and *Pink1* KO mice were cultured with the osteoclastogenic medium for 2 days. The mRNA levels of indicated genes were analyzed by RT-PCR. (D) Representative images of dentine slices. The BMMs from *Pink1* WT and *Pink1* KO were cultured on dentin slices, and the images of dentin slices were obtained by using confocal microscope. *P < 0.05, **P < 0.01. Scale bar, 75 μm. (E) *Pink1* WT and *Pink1* KO BMMs were cultured with osteoclastogenic medium for 2 days and subjected to Fluo-4/AM staining. Scale bar, 50 μm. (F) Total lysates of *Pink1* WT and *Pink1* KO BMMs cultured under osteoclastogenic conditions for 2 days were subjected to western blotting. The density of bands relative to α-Tubulin was quantified by ImageJ. *P < 0.05 (G) *Pink1* WT and *Pink1* KO pOCs were serum-starved and stimulated with RANKL (500 ng/ml) for 15 min. Representative confocal images are presented. Scale bar, 20 μm. (For interpretation of the references to color in this figure legend, the reader is referred to the Web version of this article.)

Since PINK1 deficiency aggravated periodontitis (Fig. 1), we next investigated whether PINK1 has a direct role in osteoclast differentiation and function. The loss of PINK1 expression in BMMs from *Pink1* KO mice was confirmed by RT-PCR and Western blot (Fig. 2A). The osteoclastogenic potential of *Pink1* KO BMMs was found to be higher than *Pink1* WT BMMs with regard to the number of TRAP ^+^ osteoclasts generated and the expression level of osteoclast marker genes such as *Nfatc1*, acid phosphatse 5 (*Acp5,* gene name for TRAP), ATPase H^+^ transporting V0 subunit D2 (*Atp6v0d2*), matrix metalloproteinase-9 (*Mmp9*) and dendrocyte expressed seven transmembrane protein (*Dcstamp*) (Fig. 2B and C). Consistently, *Pink1 KO* group exhibited higher bone resorption capacity in terms of resorption pit-depth and resorption area (Fig. 2D). To rule out potential indirect effects that might have changed intrinsic properties of BMMs induced by *in vivo* KO of *Pink1*, *Pink1* WT BMMs were transfected with *Pink1* siRNA and osteoclastogenesis was evaluated. Like as Pink1 KO, *Pink1* knockdown also increased the number of TRAP ^+^ cells generated, the expression of osteoclastogenic marker genes, and the extent of dentin slice resorption in osteoclast differentiation cultures (Supplementary Fig. 1). These findings indicate that PINK1 has a direct role in regulating osteoclast formation and function.

NFATc1 is a master transcription factor for osteoclast differentiation. Cytoplasmic NFATc1 translocate to the nucleus and regulates transcription of target genes, including *Acp5*, *Atp6v0d2*, *Mmp9*, and *Dcstamp*, once dephosphorylated by calcineurin. Previous studies have demonstrated that deletion of *Pink1* can induce mitochondria-to-nucleus retrograde signaling via mitochondrial Ca^2+^ efflux and Ca^2+^/calcineurin signaling [21]. As the expression levels of downstream target genes of NFATc1 were significantly upregulated upon genetic ablation or knockdown of *Pink1* (Fig. 2C and Supplementary Fig. 1C), we next explored if PINK1 deficiency could increase intracellular Ca^2+^ level and nuclear translocation of NFATc1. pOCs of *Pink1* KO group displayed an increase in cytoplasmic Ca^2+^ contents as measured with Fluo-4 Ca^2+^ indicator (Fig. 2E). The cytoplasmic phosphorylated NFATc1 (*p*-NFATc1) level was reduced whereas the total NFATc1 level was upregulated by *Pink1* KO (Fig. 2F). Moreover, nuclear translocation of NFATc1 was much higher in *Pink1* KO cells (Fig. 2G). These results suggest that PINK1 has a role in preventing excessive activation of NFATc1 to attenuate osteoclast differentiation.

### 3.3 PINK1 depletion disrupts mitochondrial morphology, decreases mitochondrial membrane potential, and increases ROS production during osteoclastogenesis

**Fig. 3:**
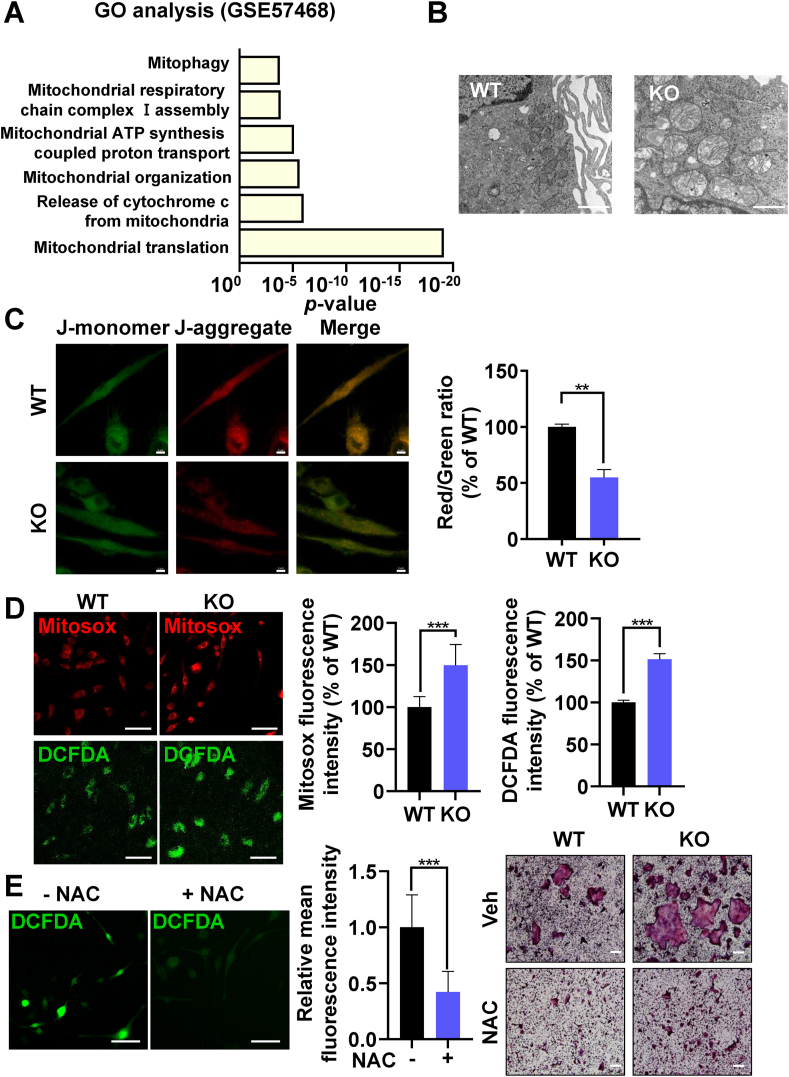
PINK1 regulates mitochondrial functions during osteoclastogenesis. (A) Enriched pathway analysis using the database of GSE57468. (B) Representative TEM images of *Pink1* WT and *Pink1* KO pOCs. Scale bar, 1 μm. (C, D) Relative levels of mitochondrial membrane potential, mitochondrial ROS, and intracellular ROS in *Pink1* WT and *Pink1* KO pOCs. **P < 0.01, ***P < 0.001. (E) BMMs treated with vehicle (Veh) or NAC (2.5 mM) in the presence of RANKL and M-CSF for 3 days were subjected to confocal analysis for intracellular ROS. ***P < 0.001 (left panel). Scale bar, 50 μm. BMMs were cultured with Veh or NAC (2.5 mM) together with RANKL and M-CSF for 3 days and then stained for TRAP (right panel). Scale bar, 200 μm. (For interpretation of the references to color in this figure legend, the reader is referred to the Web version of this article.)

To further investigate the mitochondrial mechanism by which PINK1 deficiency can induce excessive osteoclast formation, GO enrichment analysis regarding mitochondrial dynamics was performed with publicly available data obtained from BMMs undergoing osteoclastic differentiation (GSE57468; [22]). Significant changes in several mitochondrial pathways, including mitophagy, were identified to occur during osteoclast differentiation (Fig. 3A). We next performed TEM to compare mitochondrial morphology of pOCs from *Pink1* WT and *Pink1* KO mice. *Pink1* KO pOCs showed disruptive features in mitochondrial organization with loss of cristae, enlarged vacuoles in matrix, and mitochondrial swellings (Fig. 3B). Despite these defects, PINK1 deficiency exerted little effects on mitochondria-mediated apoptosis (Supplementary Fig. 2).

Given that a decrease in ΔΨm favors Ca^2+^ efflux [23] and *Pink1* deletion in pOCs led to an increase in intracellular Ca^2+^ content (Fig. 2E), we assessed ΔΨm by JC-1 analyses in which red-to-green ratio indicates the state of ΔΨm. *Pink1* KO pOCs displayed reduced red fluorescence in mitochondria compared to *Pink1* WT pOCs whereas green fluorescence intensity was similar in both *Pink1* WT and *Pink1* KO groups, indicating that ΔΨm was lowered by PINK1 deficiency (Fig. 3C). A burst release of ROS from damaged mitochondria can elicit intracellular redox instability [24]. Therefore, we next measured mitochondrial and intracellular ROS levels by fluorescence intensities of Mitosox and DCFDA, respectively. Both mitochondrial and intracellular ROS levels were found out to be significantly increased in *Pink1* KO cells than those in *Pink1* WT cells (Fig. 3D). Under physiological condition, ROS function as a second messenger for osteoclast differentiation. Thus, we investigated if hyperactivation of osteoclast differentiation in *Pink1* KO cells could be attenuated by scavenging excess ROS with NAC. NAC treatment suppressed the excessive generation of mature osteoclasts from *Pink1* KO cells by reducing ROS level as shown by fluorescence intensity of DCFDA (Fig. 3E). Overall, these results suggest that changes in ΔΨm and mitochondrial ROS production upon genetic deletion of *Pink1* lead to a higher propensity for osteoclast differentiation.

### 3.4 Mitochondrial oxygen consumption is decreased but overall ATP production is sustained in Pink1 KO osteoclasts

**Fig. 4:**
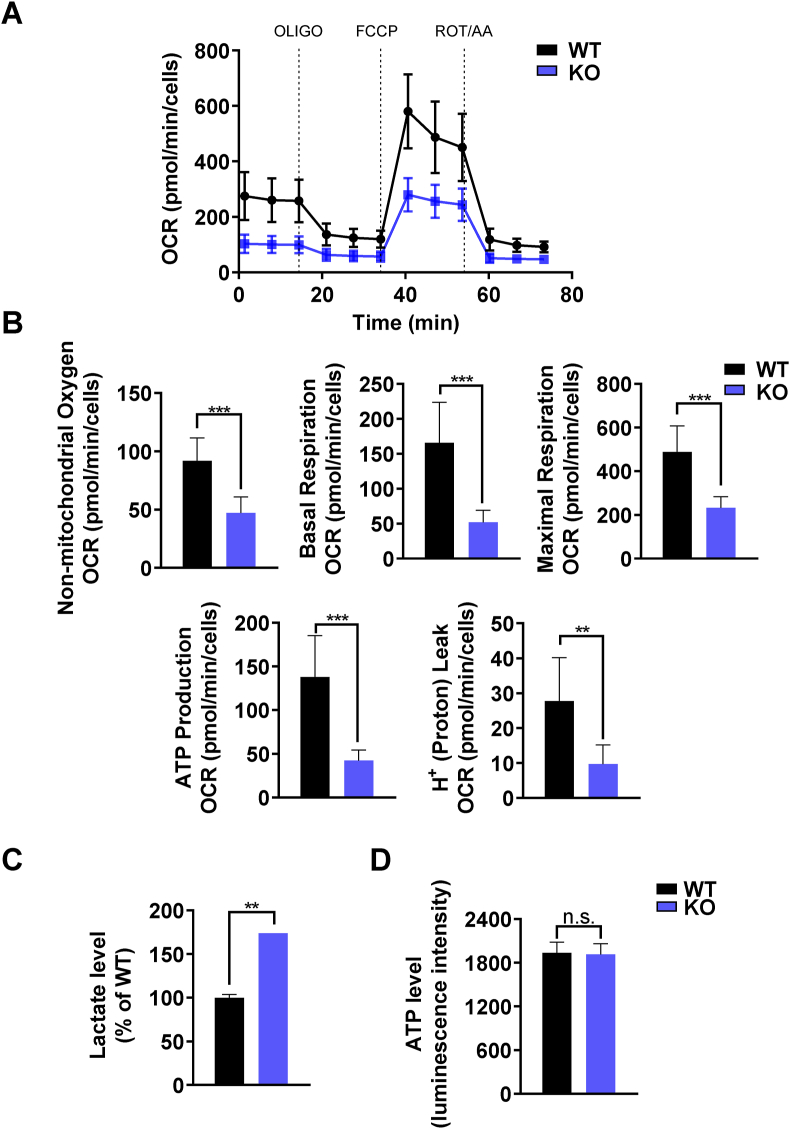
PINK1 deficiency decreased OCR. (A) The OCR from *Pink1* WT and *Pink1* KO pOCs was assessed using the Seahorse XF96 analyzer. (B) Data were analyzed by using the Wave 2.6.1 software (*n* = 8). Bar charts represent mean ± SD. **P < 0.01, ***P < 0.001. (C). Intracellular lactate levels of *Pink1* WT and *Pink1* KO pOCs were measured by using a colorimetric/fluorometric assay kit. **P < 0.01 versus WT. (D) Intracellular ATP levels of *Pink1* WT and *Pink1* KO pOCs were measured by using a luminescent ATP assay kit. n.s., non-significant. (For interpretation of the references to color in this figure legend, the reader is referred to the Web version of this article.)

Mitochondrial dysfunction is often associated with decrease in mitochondrial respiration. Therefore, we hypothesized that the accumulation of damaged mitochondria alters bioenergetic state in *Pink1* KO pOCs. To investigate the effects of PINK1 in mitochondrial respiration during osteoclast differentiation, we employed the Seahorse XF Cell Mito Stress Test. The basal respiration is measured by subtracting non-mitochondrial respiration from baseline OCR. ATP production OCR and H^+^ leak OCR were obtained through inhibition of complex V by treatment of oligomycin (OLIGO). FCCP was treated to induce mitochondrial uncoupling to measure maximal respiratory OCR. Lastly, rotenone A and antimycin A (ROT/AA) were used to stop electron transport chain for non-mitochondrial respiration measurements. The mitochondrial respiration was disrupted in *Pink1* KO pOCs compared to *Pink1* WT pOCs. Non-mitochondrial oxygen, basal respiration, maximal respiration, ATP production and proton leak OCR were significantly decreased in *Pink1* KO pOCs compared to *Pink1* WT pOCs (Fig. 4A and B). Osteoclasts, on the other hand, require large amounts of ATP for bone resorption [25]. It has been reported that loss of *Pink1* in primary mouse embryonic fibroblasts stimulated glucose uptake and lactate release [19]. Similarly, lactate levels of *Pink1* KO pOCs was increased compared to *Pink1* WT pOCs (Fig. 4C) and results of ECAR analyses indicated enhanced glycolysis in *Pink1* KO pOCs (Supplementray Fig. 3). However, the total ATP level was comparable between two groups (Fig. 4D). These results demonstrate that loss of PINK1 in pOCs reduces mitochondrial respiration, but sufficient amount of ATP for osteoclast differentiation is sustained, due to an increase in glycolysis in *Pink1* KO osteoclasts.

### 3.5 Mitophagy is suppressed in Pink1 KO osteoclasts

**Fig. 5:**
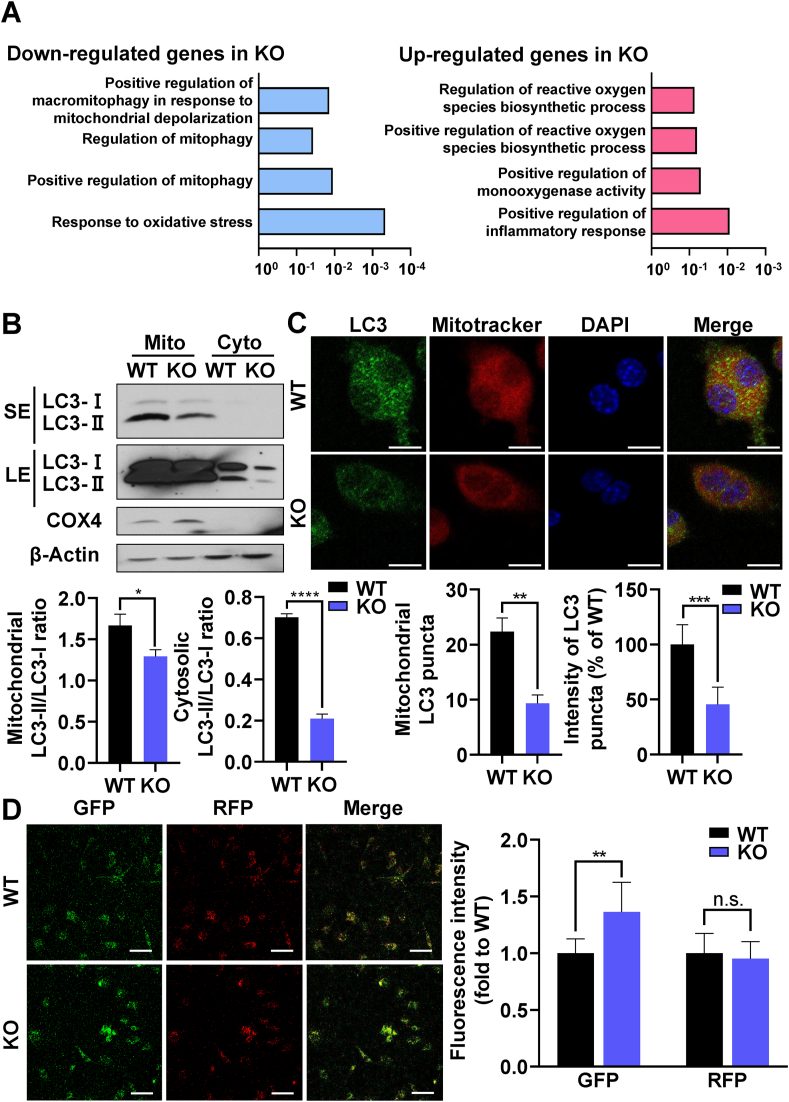
PINK1 deficiency promotes osteoclastogenesis by regulating mitophagy. (A) RNA sequencing of *Pink1* WT and *Pink1* KO pOCs was performed and GO functional clusters of up- and down-regulated genes were presented. The length of the bar represents the -log 10-transformed P-value. (B) Representative immunoblots of mitochondrial and cytosolic LC3 of *Pink1* WT and *Pink1* KO pOCs. The density of bands relative to β-Actin or COX4, were quantified by using ImageJ. *P < 0.05, ****P < 0.0001. SE, short exposure. LE, long exposure. (C) Representative confocal images of LC3 and Mitotracker staining. The number of mitochondrial LC3 puncta and the intensity of mitochondrial LC3 puncta were quantified by using ImageJ. **P < 0.01, ***P < 0.001. Scale bar, 10 μm. (D) *Pink1* WT and *Pink1* KO BMMs were transfected with the mRFP-GFP-LC3 plasmid. After 2 days of culture in the osteoclastogenic medium, cells were subjected to confocal microscopy. Scale bar, 50 μm. n.s., non-significant. (For interpretation of the references to color in this figure legend, the reader is referred to the Web version of this article.)

PINK1 promotes selective degradation of damaged mitochondria termed as mitophagy to protect cells from oxidative stress [26,27]. To gain insights on the role of PINK1 regulation of mitochondria in osteoclastogenesis, we performed quantum RNA sequencing of pOCs from *Pink1* WT and *Pink1* KO mice (Supplementary Fig. 4 and Supplementary Table 2). GO analysis of the obtained data revealed that genses involved in mitophagy were down-regulated in *Pink1* KO cells (Fig. 5A). We next evaluated mitophagy by immunoblotting and confocal analyses of LC3, a marker for autophagy, in mitochondria. Cytochorome c oxidae subunit 4 (COX4) in the Western blot was used as the loading control of mitochondrial proteins. The mitochondrial LC3 puncta and the intensity of LC3 puncta were determined (Supplementary Fig. 5). Although the expression level of LC3 in the Western blot as well as the number of LC3 puncta in confocal assay were not completely lost in *Pink1* KO cells, they were significantly decreased in the mitochondrial compartment of *Pink1* KO group compared with *Pink1* WT group (Fig. 5B and C). These data suggest that PINK1-dependent mitophagy is attenuated by PINK1 deficiency during OC differentiation.

Upon the observation of significantly reduced mitophagy in *Pink1* KO pOCs, we further investigated the effect of PINK1 deficiency on autophagolysosome formation by transfecting *Pink1* WT and *Pink1* KO cells with tandem-tagged RFP-GFP-LC3, which displays yellow fluorescent puncta in autophagosomes and red puncta in autophagolysosome as GFP fluorescence is quenched off in acidic autophagolysosome. A greater intensity of green fluorescence was observed in *Pink1* KO than in *Pink1* WT pOCs, suggesting that PINK1 deficiency led to impairments in autophagolysosome formation (Fig. 5D). Taken together, these results suggest that PINK1-deficiency leads to reduced mitophagy as a part of the overall autophagy during osteoclast differentiation.

### 3.6 SPD treatment attenuates excessive osteoclastogenesis induced by PINK1 deficiency

**Fig. 6:**
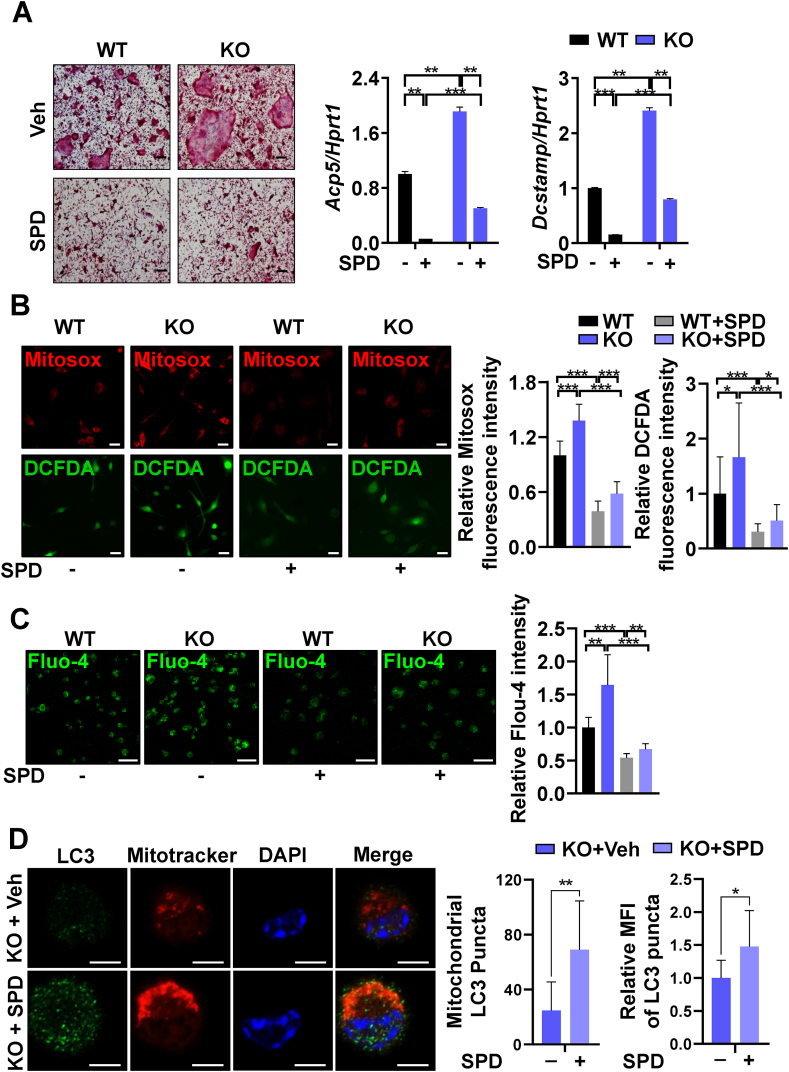
Induction of mitophagy and autophagy attenuates PINK1 deficiency-induced enhancement of osteoclastogenesis and mitochondria damage. (A) *Pink1 WT* and *Pink1* KO BMMs were cultured with or without SPD (5 μM) in the osteoclastogenic medium, and subjected to TRAP staining and RT-PCR. (B) Representative confocal images for mitochondrial and intracellular ROS of *Pink1* WT and *Pink1* KO pOCs treated with or without SPD (5 μM). Scale bar, 20 μm. (C) *Pink1* WT and *Pink1* KO pOCs treated with or without SPD (5 μM) were subjected to Fluo-4/AM staining for intracellular Ca^2+^. Scale bar, 50 μm. (D) *Pink1* KO BMMs were cultured with or without SPD (5 μM) in the osteoclastogenic medium for 2 days and the cells were subjected to confocal microscopy. The number of mitochondrial LC3 puncta and the intensity of mitochondrial LC3 puncta were quantified by using ImageJ. *P < 0.05, **P < 0.01. Scale bar, 5 μm. (For interpretation of the references to color in this figure legend, the reader is referred to the Web version of this article.)

SPD, a natural polyamine that can induce mitophagy and autophagy [28], was shown to prevent bone loss in ovariectomy-induced osteoporosis mice [29]. Similarly, osteoclast differentiation was suppressed by SPD treatment in a dose-dependent manner (Supplementary Fig. 6A). The expression of NFATc1 was also downregulated by SPD (Supplementary Fig. 6B). Furthermore, SPD treatment upregulated both mitophagy and autophagy in pOCs, as revealed by total and mitochondria-associated LC3 puncta in confocal analyses (Supplementary Fig. 6C). Thus, we hypothesized that SPD may inhibit excessive osteoclast formation caused by genetic ablation of *Pink1*. The addition of SPD blunted the effects of PINK1 deficiency on osteoclast formation and the expression of NFATc1 downstream target genes, *Acp5* and *Dcstamp* (Fig. 6A). Consistently, SPD treatment significantly relieved mitochondrial and intracellular oxidative stress as well as intracellular Ca^2+^ overloads in *Pink1* KO cells as shown by confocal microscopy of Mitosox, DCFDA, and Fluo-4 (Fig. 6B and C). Then, we evaluated the effects of SPD treatment on mitophagy in *Pink1* KO cells. As a result, SPD treatment increased the number and intensity of mitochondrial LC3 puncta, as well as overall LC3 expression, in *Pink1 KO* cells (Fig. 6D). These results indicate that upregulation of mitophagy and autophagy through SPD treatment alleviates mitochondrial dysfunction caused by depletion of *Pink1*, and consequently suppresses the excessive osteoclast differentiation from *Pink1* KO BMMs.

## 4 Discussion

Maintaining mitochondrial homeostasis is an essential means to restrain exorbitant osteoclast formation [8]. However, the function of PINK1, a well-known mitophagy inducer, in osteoclast differentiation and bone resorption has not been clearly investigated. Here, we showed that PINK1 deficiency exacerbated periodontitis by promoting inordinate osteoclast formation (Fig. 1). The genetic deletion of *Pink1* led to mitochondrial defects, enhanced nuclear translocation of NFATc1, an excessive ROS production, and impaired mitochondrial respiration in osteoclasts (Fig. 2, Fig. 3, Fig. 4). In addition, PINK1 deficiency showed reduced mitophagy and impaired autophagic flux (Fig. 5). These results are in line with the view that mitophagy, a mitochondrial quality control mechanism, may prevent age-related diseases [30] by putting a rein to a burst of Ca^2+^ release from mitochondria and to an irritative ROS production [23,31]. Under hypoxic condition, mitochondrial ROS upregulates osteoclast differentiation, which can be resolved upon antioxidant treatment [32]. In compliance with PINK1 deficiency, the mitochondrial and intracellular ROS levels were massively increased in *Pink1* KO cells (Fig. 3D), but the resolution of oxidative stress via NAC treatment efficiently reduced osteoclast generation (Fig. 3E). Furthermore, the overactivation of osteoclast differentiation and mitochondrial impairments due to genetic ablation of *Pink1* was apparently resolved upon stimulation of mitophagy and autophagy by SPD treatment (Fig. 6). Collectively, these findings suggest that PINK1 has an explicit function in maintaining mitochondrial homeostasis, limiting excessive osteoclast formation, and subsequently retaining intact periodontal health.

Clinical and animal studies have consistently suggested that gingival infiltration of classically activated macrophage (M1) macrophage is inevitable in periodontal disease [33]. The cGAS-STING pathway, which is frequently found to be stimulated in periodontitis [34], may not only induce type I interferon responses but also promote M1 polarization by sensing cytosolic DNA [35]. Recently, Sliter and colleagues showed that the loss of PINK1 expression may result in mitochondrial DNA leakage into cytosol, promoting activation of the cGAS/STING pathway [36]. Thus, it cannot be excluded that genetic deletion of *Pink1* may exert detrimental effects on periodontitis via M1 polarization through the cGAS-STING pathway. In addition, M1 macrophages can also secrete pro-inflammatory cytokines such as IL-1, IL-6, and TNFα that may influence osteoclastogenesis. Therefore, PINK1 deficiency may indirectly affect the osteoclast differentiation under *in vivo* pathological conditions. Although, its role in M1 polarization in periodontitis requires further investigation, here we clearly showed a direct role of PINK1 in osteoclast differentiation and function.

Currently, microbial dysbiosis and overactivation of innate and adaptive immune systems are accounted for the main causes of periodontitis [37]. Given that PINK1 deficiency has been reported to facilitate the formation of NLR family pyrin domain containing protein 3 (NLRP3) inflammasome in bone marrow-derived macrophages [38,39] and NLRP3 is closely associated with the differentiation of osteoclasts in the alveolar bone [40], systemic ablation of *Pink1* may worsen the inflammatory status of experimental periodontitis in virtue of overactivation of NLRP3 inflammasome. Besides, the activation of NLRP3 inflammasome is commonly observed in patients with chronic periodontitis [41]. Therefore, we could not rule out the effects of PINK1 deficiency on innate immune responses in ligature-induced periodontitis. On the other hand, with respect to NLRP3 inflammasome-related alveolar bone loss, the age factor should be taken into consideration as it was significantly suppressed only in aged mice but not in young mice upon inhibition of NLRP3 inflammasome by MCC950 treatment in the mouse model of LPS-induced periodontitis [42]. In line with this, our *in vitro* study demonstrated that PINK1 deficiency can directly increase osteoclast differentiation and bone resorption in BMMs from young mice; yet, the effects of PINK1 with respect to an age factor on the formation of NLRP3 inflammasome and its contribution to the alveolar bone loss in our model warrant further studies.

The importance of PINK1 for mitophagic flux during osteoclastogenesis was evidenced by the results of Western blot and confocal analyses of mitochondrial LC3 (Fig. 5B). The contribution of PINK1 on overall autophagy in osteoclasts was assessed by examining LC3-GFP-RFP (Fig. 5C). The greater GFP fluorescence in the *Pink1* KO group (Fig. 5C) suggests that autophagic flux is suppressed by PINK1 deficiency. This notion was further corroborated by the result of SPD treatment. SPD that stimulates mitophagy and autophagy nullified the effect of PINK1 deficiency on osteoclast generation (Fig. 6A∼D), supporting that the PINK1 deficiency-induced excessive osteoclastogenesis was due to the reduction of overall autophagy including mitophagy. Mitophagy can be mediated by both PINK1-dependent and PINK1-independent mechanisms. To date, mitochondrial E3 ubiquitin ligase 1-dependent mitophagy, NIP3-like protein X or FUN14 domain containing 1 receptor-mediated mitophagy, and cardiolipin-mediated mitophagy have been identified in addition to PINK1-dependent mitophagy [43]. Although we could not specify which compensatory mitophagy was occurred in SPD-treated *Pink1* KO cells, mitochondrial LC3 puncta were found to be increased (Fig. 6D). In addition, the expression of LC3 and the formation of LC3 puncta were not completely lost in the mitochondria of *Pink1* KO cells (Fig. 5B). Therefore, the alternative mitophagies or general autophagy may be operated in the *Pink1* KO cells and SPD may augment these compensatory pathways to remove defective mitochondria during osteoclast differentiation.

A limitation of this study is that the effect of PINK deficiency on mitophagy could not be distinguished from that on mitochondrial abnormality. As PINK1 has both mitophgy-dependent and mitophagy-independent roles, whether PINK1 also regulates mitochondria quality in a mitophagy-independent way during osteoclastogenesis is a question to be resolved. If both mechanisms are operated in PINK1-mediated regulation of osteoclasts and bone homeostasis, although we have shown that SPD may mitigate *Pink1* KO-induced abnormal osteoclastogenesis possibly by stimulating PINK1-independent mitophagy (Fig. 6D), the proportion of contribution by mitophagy needs to be further addressed. Nevertheless, our findings indicate that PINK1 is essential for regulating mitochondrial quality during osteoclast differentiation, whereby loss of PINK1 can lead to mitochondrial abnormalities, followed by excessive osteoclastogenesis and exacerbation of periodontitis.

In this study, we demonstrated that PINK1 controls inappropriate formation of osteoclasts that is frequently found in periodontal diseases. Meta-analysis of osteoporotic patients indicated that mitochondrial dysfunction is associated with osteoporosis [44]. In addition, significant reduction of PINK1 expression was found in the bone of ovariectomized model mice [45]. We showed that SPD, a natural mitophagy inducer, could mitigate detrimental effects caused by genetic ablation of *Pink1*, thereby attenuate excessive osteoclast differentiation. Taken together, targeting PINK1 to prevent overstimulation of osteoclastogenesis may provide a novel therapeutic strategy not only for periodontitis but also for other age-associated skeletal diseases.

## 5 Conclusions

In conclusion, our study revealed for the first time the importance of PINK1 in controlling the extent of osteoclastogenesis. PINK1 mediates the removal of defective mitochondria through mitophagy, which prevents excessive ROS stress and Ca^2+^ overload, restraining overstimulation of osteoclast differentiation. We also verified the *in vivo* role of PINK1 in osteoclastogenesis control by experiments with a periodontitis-associated bone loss model. Our findings signify PINK1 as a novel player in osteoclastogenesis and as a potential therapeutic target against bone lytic diseases.

## Funding

This work was supported by the National Research Foundation of Korea (NRF) grant funded by the Korea government (MSIT) (RS-2023-00252102, NRF-2020R1A2C2010082, and NRF-2018R1A5A2024418) to H-HK.

## Authorship contribution statement

**Ji Sun Jang:** Conceptualization, Data curation, Formal analysis, Investigation, Writing – original draft, Writing – review & editing. **Seo Jin Hong:** Conceptualization, Data curation, Formal analysis, Investigation, Writing – original draft, Writing – review & editing. **Shenzheng Mo:** Data curation, Formal analysis, Writing – review & editing. **Min Kyung Kim:** Conceptualization, Supervision, Writing – review & editing. **Yong-Gun Kim:** Formal analysis, Methodology, Writing – review & editing. **Youngkyun Lee:** Formal analysis, Methodology, Writing – review & editing. **Hong-Hee Kim:** Conceptualization, Data curation, Formal analysis, Funding acquisition, Project administration, Supervision, Writing – original draft, Writing – review & editing.

## Declaration of competing interest

The authors declared no potential conflicts of interest with respect to the research, authorship, and/or publication of this article.

## Data Availability

Data will be made available on request.
