# Supplementary material for: PINK1 restrains periodontitis-induced bone loss by preventing osteoclast mitophagy impairment
Source: Redox Biol. 2023 Dec 30;69:103023. doi: 10.1016/j.redox.2023.103023 (PMC10789640; doi:10.1016/j.redox.2023.103023)
Supplement: Multimedia component 1 [file mmc1.docx]

**Supplementary information**

**PINK1 restrains periodontitis-induced bone loss by preventing osteoclast mitophagy impairment**

Ji Sun Jang ^a,1^, Seo Jin Hong ^a,1^, Shenzheng Mo ^a^, Min Kyung Kim ^a^, Yong-Gun Kim ^b^, Youngkyun Lee ^c^, and Hong-Hee Kim^a,*^

^1^These authors contributed equally to this article as the co-first authors.

^a^Department of Cell and Developmental Biology, Dental Research Institute, School of Dentistry, Seoul National University, Seoul 03080, Republic of Korea

^b^Department of Periodontology, School of Dentistry, Kyungpook National University, Daegu 41940, Republic of Korea

^c^Department of Biochemistry, School of Dentistry, Kyungpook National University, Daegu 41940, Republic of Korea

**Corresponding author*: Hong-Hee Kim, Department of Cell and Developmental Biology, School of Dentistry, Seoul National University, 101 Daehak-ro, Jongno-gu, Seoul 03080, Korea. E-mail: hhbkim@snu.ac.kr

**Materials and Methods**

**Genetic knockdown of BMMs**

BMMs were transfected with HiPerFect (Qiagen, Hilden, Germany) containing 30 nM of *Pink1* small interfering RNA (siRNA) oligonucleotides (Bioneer, Daejoen, Korea) for 6-14 h. The transfection medium was replaced with α-MEM complete medium containing 100 ng/ml of RANKL and 30 ng/ml of M-CSF, and cells were cultured for indicated days.

**Flow cytometric analysis of apoptosis**

The apoptosis of pOCs was analyzed by flow cytometry with 1x10^4^-cell readouts by using Annexin V-FITC/ Propidium iodide (PI) apoptosis kit (Becton, CA, USA) and LSR-Fortessa X-20 (Becton) according to manufacturer’s guidelines. Data were assessed by using Flowzo software (Becton).

**Supplementary Figure 1.**

**
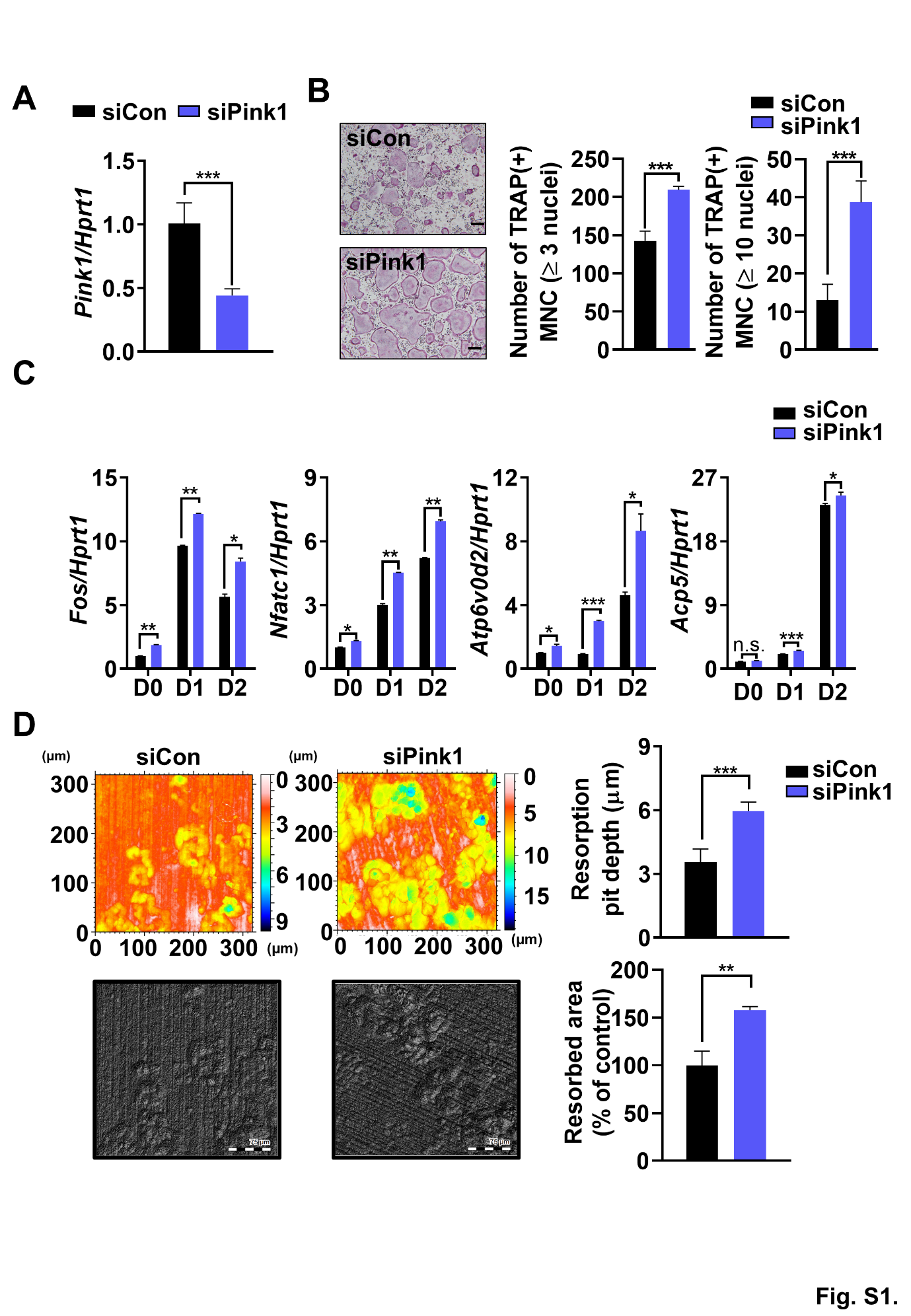
**

**Supplementary Figure 1.** PINK1 knockdown enhances osteoclast differentiation. (A) RT-PCR shows a significant decrease of *Pink1* mRNA in PINK1 knockdown osteoclasts. ****P* < 0.001. (B, C) BMMs treated with control and *Pink1* siRNA were cultured with osteoclastogenic medium and subjected to (B) TRAP staining and (C) RT-PCR **P* < 0.05, ***P* < 0.01, ****P* < 0.001 versus the control knockdown group. MNC, multinucleated cells. (D) Representative images of dentine slices on which *Pink1* knockdown and control knockdown osteoclasts were cultured were taken using a confocal microscope. ***P* < 0.01, ****P* < 0.001. Scale bar, 75 μm.

**Supplementary Figure 2.**


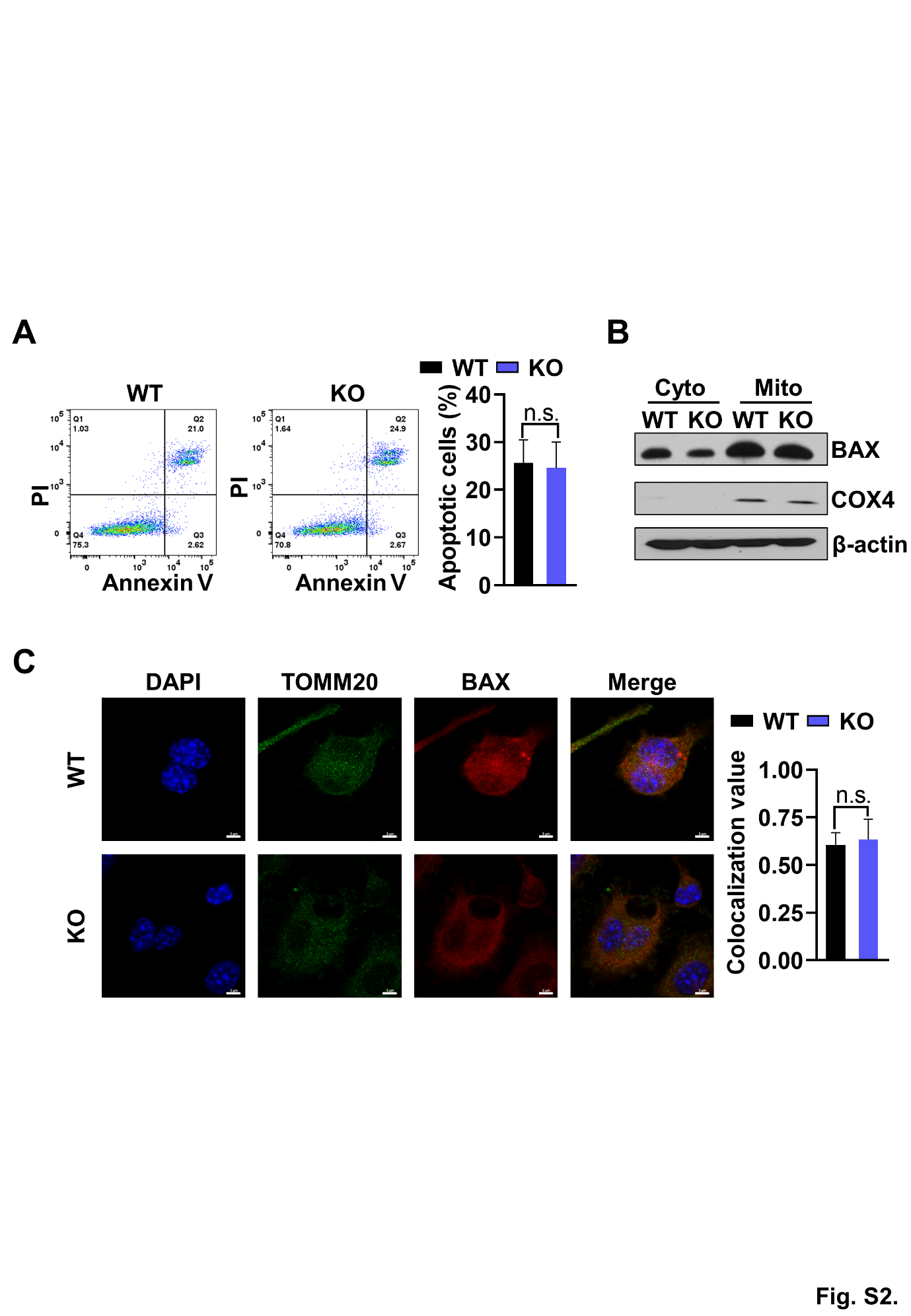


**Supplementary Figure 2.** No significant difference in apoptosis between *Pink1 WT* and *Pink1* KO pOCs. (A) BMMs from *Pink1 WT* and *Pink1* KO mice were cultured with an osteoclastogenic medium for 2 days. The degree of apoptosis was determined by Annexin V/PI staining followed by flow cytometry. (B, C) *Pink1 WT* and *Pink1* KO BMMs were cultured in the presence of RANKL and M-CSF for 2 days and subjected to (B) western blotting and (C) confocal microscopy. Cyto, cytosol. Mito, mitochondria.

**Supplementary Figure 3.**

**
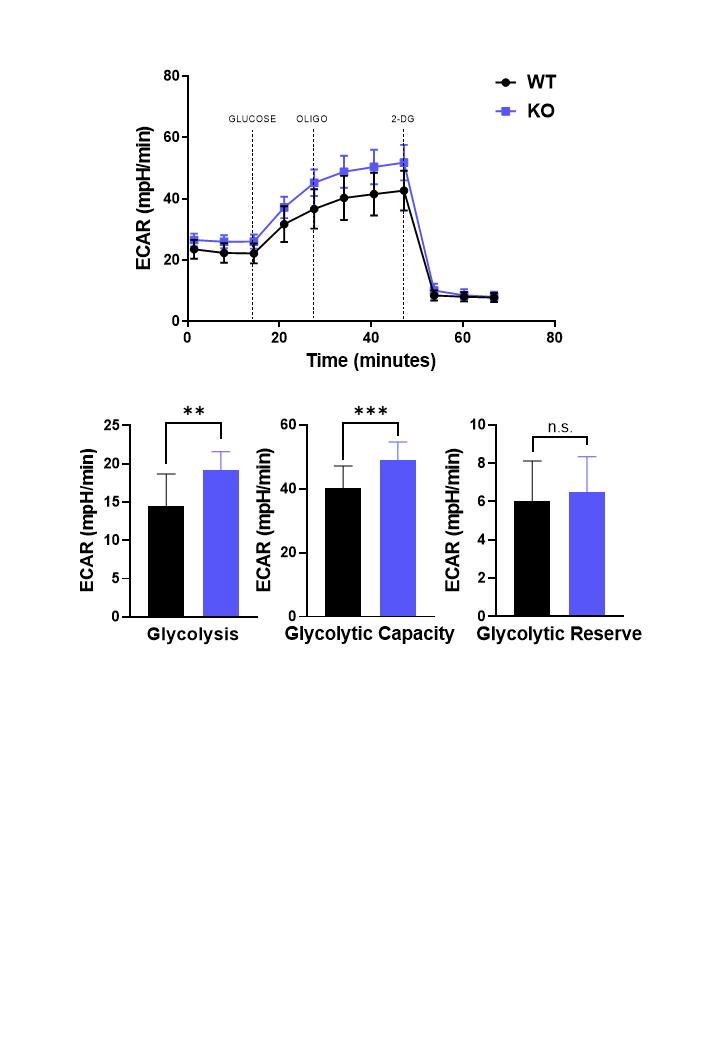
Supplementary Figure 3.** PINK1 deficiency increases glycolysis and glycolytic capacity. ECAR for *Pink1* WT and *Pink1* KO pOC was assessed using Seahorse XF96 system. The glycolysis, glycolytic capacity, and glycolytic reserve were analyzed with Wave 2.6.1 software (*n* = 11). Bar charts represent mean ± SD. ***P* < 0.01, ****P* < 0.001.

**Supplementary Figure 4.**


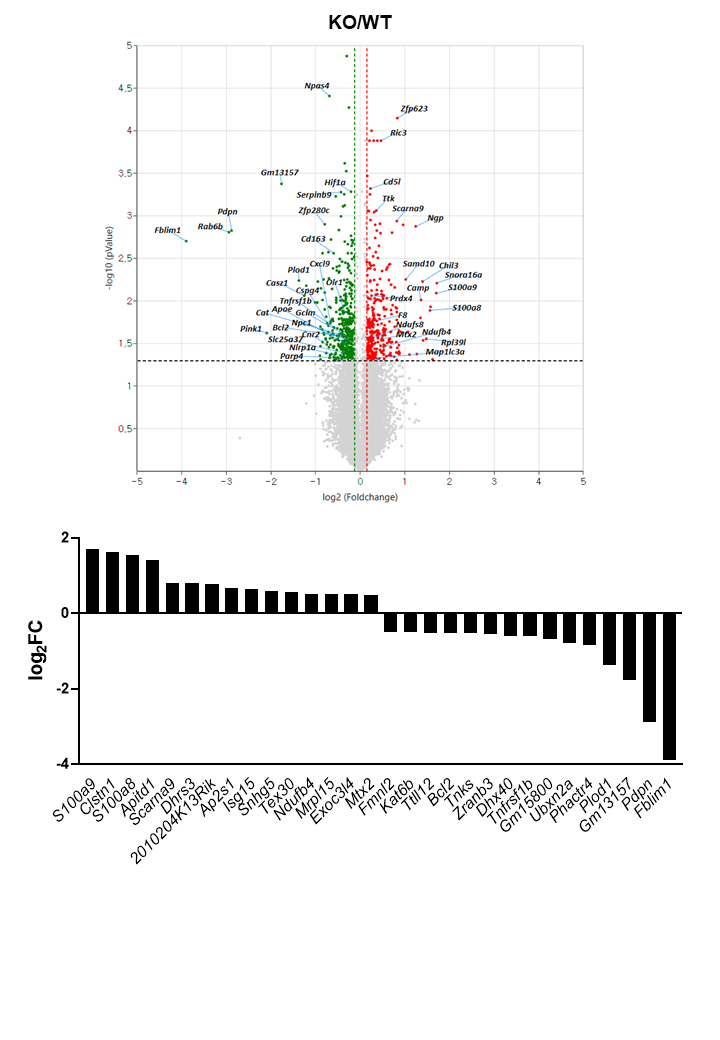

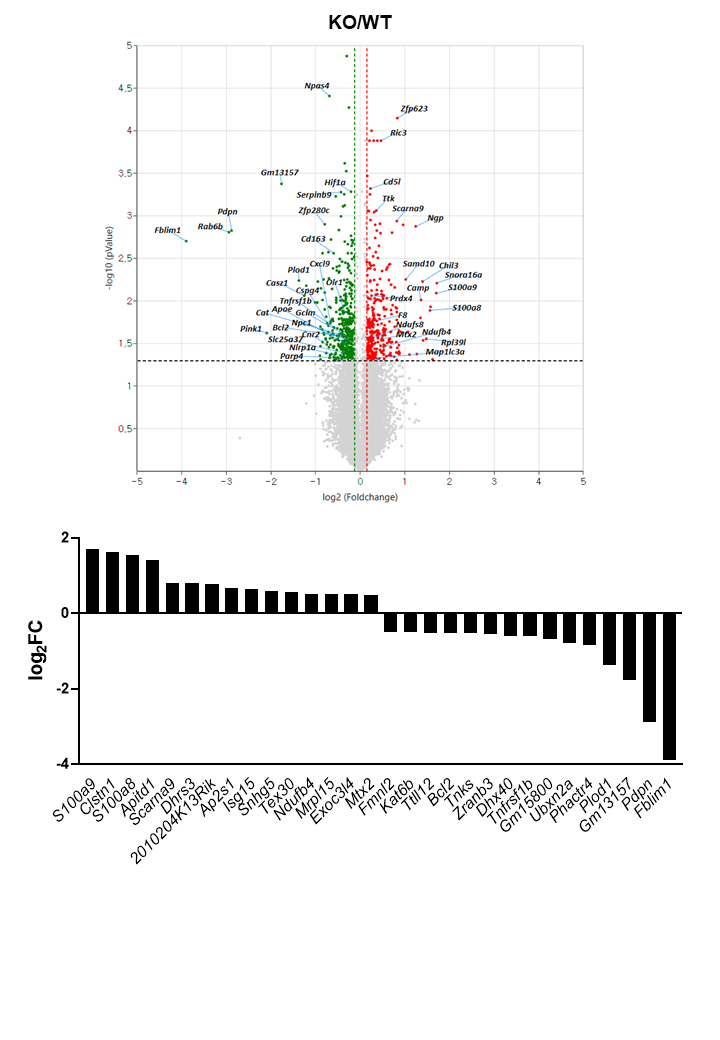


KO/WT

**Supplementary Figure 4.** DEGs of *PINK1* KO pOCs. The volcano plot and the bar graph of genes up-regulated and down-regulated with significant changes in *Pink1* KO in comparison *Pink1* WT cells are shown.

**Supplementary Figure 5.**


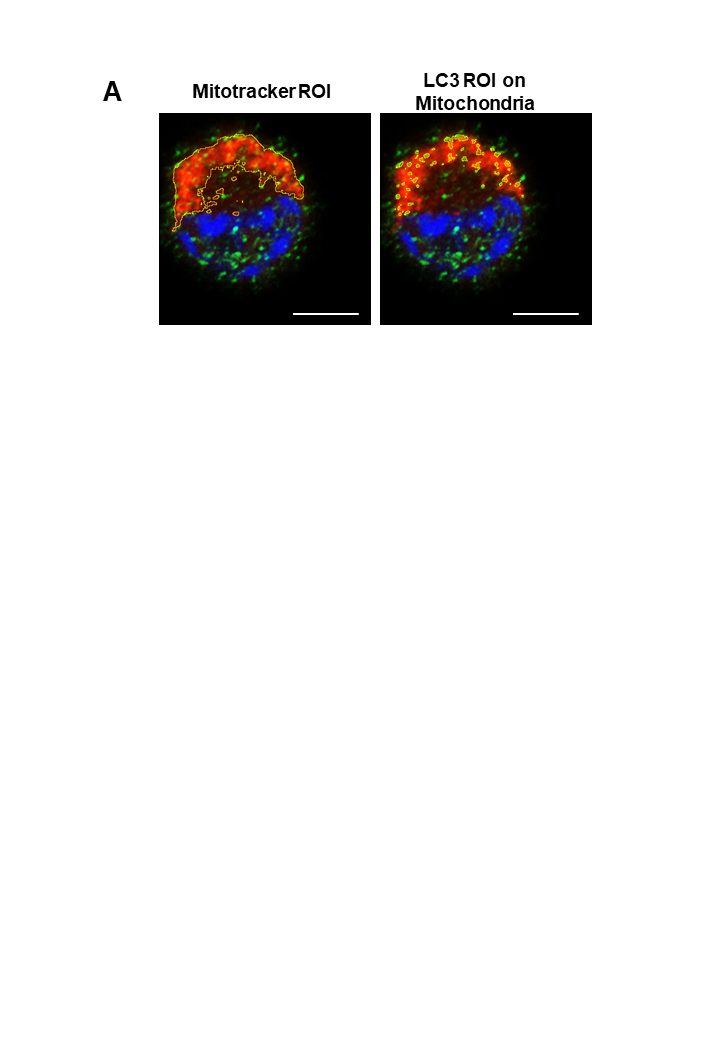


**Supplementary Figure 5.** Determination of ROIs of mitochondria and of LC3 puncta within the mitochondria. The ImageJ software was used to determine respective ROIs with certain thresholds of fluorescence intensities. Scale bar, 5μm.

**Supplementary Figure 6.**

**
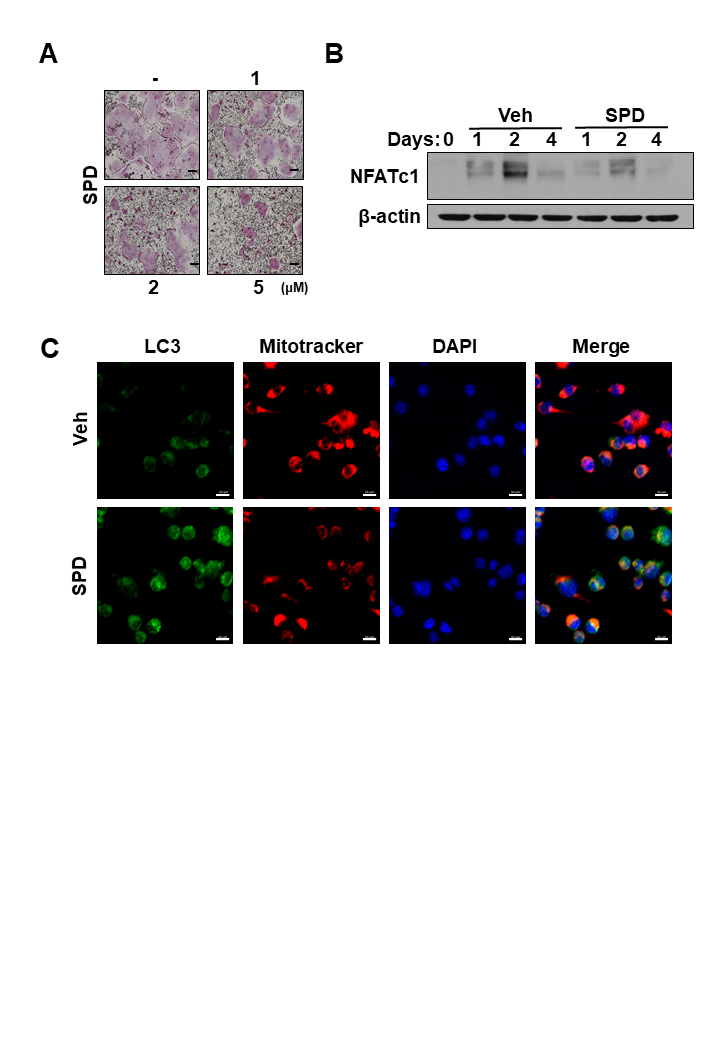
**

**Supplementary Figure 6.**  SPD induces mitophagy and inhibits osteoclast differentiation. (A, B) BMMs cultured in the osteoclastogenic condition in the presence of different concentrations of SPD were subjected to (A) TRAP staining and (B) western blotting. Scale bar, 200 μm. (C) BMMs treated with vehicle and SPD (5 μM) were cultured with osteoclastogenic medium for 2 days and subjected to confocal microscopy. Scale bar, 20 μm.

**Supplementary Table 1.** Primer sequences for gene expression used in this study.

| Gene | Sequence |
| --- | --- |
| **mPink1** | Forward: 5′ -TGAGGAGCAGACTCCCAGTT |
|  | Reverse: 5′ -CTTGAGATCCCGATGGGCAA |
| **mAcp5** | Forward: 5′ -CGACCATTGTTAGCCACATACG |
|  | Reverse: 5′ -TCGTCCTGAAGATACTGCAGGTT |
| **mAtp6v0d2** | Forward: 5′ -GGGAGACCCTCTTCCCCACC |
|  | Reverse: 5′ -CCACCGACAGCGTCAAACAAA |
| **mNfatc1** | Forward: 5′ -CCAGTATACCAGCTCTGCCA |
|  | Reverse: 5′ -GTGGGAAGTCAGAAGTGGGT |
| **mfos** | Forward: 5′ -ACTTCTTGTTTCCGGC |
|  | Reverse: 5′ -AGCTTCAGGGTAGGTG |
| **mMmp9** | Forward: 5'-GACGGCACGCCTTGGTGTAG |
|  | Reverse: 5’-AGGAGCGGCCCTCAAAGATG |
| **mDcstamp** | Forward: 5’-GGGTGCTGTTTGCCGCTG |
|  | Reverse: 5’-CGACTCCTTGGGTTCCTTGCT |
| **mHprt1** | Forward: 5′ -CCTAAGATGAGCGCAAGTTGAA |
|  | Reverse: 5′ -CCACAGGGACTAGAACACCTGCTAA |

**Supplementary Table 2.** List of genes up-regulated and down-regulated in *Pink1* KO.

| Up-regulated gene list | | | | | | | | |
| --- | --- | --- | --- | --- | --- | --- | --- | --- |
|  |  |  |  |  |  |  |  |  |
| **Gene ID** | **Symbol** | ***p* value** | **Fold Change** |  | **Gene ID** | **Symbol** | ***p* value** | **Fold Change** |
| 14669 | *Snora16a* | 0.006 | 3.291 |  | 4917 | *Prl2c2* | 0.023 | 1.609 |
| 13104 | *S100a9* | 0.008 | 3.255 |  | 15536 | *Shisa3* | 0.013 | 1.606 |
| 14983 | *Clstn1* | 0.049 | 3.062 |  | 17968 | *Ap2s1* | 0.023 | 1.600 |
| 14964 | *Angptl7* | 0.012 | 2.983 |  | 1928 | *4930404N11Rik* | 0.035 | 1.597 |
| 13103 | *S100a8* | 0.013 | 2.940 |  | 20951 | *Kbtbd3* | 0.041 | 1.595 |
| 7523 | *Rpl39l* | 0.028 | 2.775 |  | 22685 | *Haus7* | 0.004 | 1.586 |
| 14974 | *Apitd1* | 0.029 | 2.648 |  | 15071 | *Atad3aos* | 0.028 | 1.583 |
| 13371 | *Chil3* | 0.006 | 2.628 |  | 15096 | *Isg15* | 0.030 | 1.577 |
| 22083 | *Camp* | 0.010 | 2.560 |  | 17416 | *P3h3* | 0.018 | 1.576 |
| 3590 | *Snora21* | 0.016 | 2.542 |  | 9794 | *Mppe1* | 0.028 | 1.562 |
| 9993 | *D330050I16Rik* | 0.042 | 2.400 |  | 6258 | *Gm5801* | 0.043 | 1.559 |
| 22092 | *Ngp* | 0.001 | 2.359 |  | 21875 | *Gk5* | 0.029 | 1.559 |
| 18033 | *Klc3* | 0.043 | 2.136 |  | 6958 | *Slc39a4* | 0.006 | 1.555 |
| 16133 | *Snora15* | 0.023 | 2.088 |  | 8718 | *H2-Oa* | 0.004 | 1.553 |
| 12592 | *Samd10* | 0.006 | 2.032 |  | 16320 | *Papolb* | 0.004 | 1.522 |
| 6422 | *9930012K11Rik* | 0.001 | 1.937 |  | 16516 | *1110019D14Rik* | 0.031 | 1.517 |
| 13753 | *Triqk* | 0.023 | 1.881 |  | 12449 | *Atp9a* | 0.009 | 1.514 |
| 14096 | *Bspry* | 0.041 | 1.831 |  | 21832 | *Snhg5* | 0.044 | 1.494 |
| 5931 | *Mustn1* | 0.042 | 1.830 |  | 14937 | *Rex2* | 0.004 | 1.492 |
| 15257 | *4831440E17Rik* | 0.022 | 1.822 |  | 3913 | *Sox9* | 0.013 | 1.492 |
| 11222 | *Erich2* | 0.039 | 1.814 |  | 7650 | *2510002D24Rik* | 0.022 | 1.483 |
| 22807 | *Spin4* | 0.033 | 1.813 |  | 9375 | *Abhd3* | 0.033 | 1.475 |
| 8554 | *Ggnbp1* | 0.041 | 1.810 |  | 22843 | *Tex11* | 0.025 | 1.474 |
| 6924 | *Zfp623* | 0.000 | 1.778 |  | 208 | *Tex30* | 0.050 | 1.469 |
| 18986 | *Kctd14* | 0.035 | 1.773 |  | 21175 | *Prdm10* | 0.023 | 1.464 |
| 4916 | *Prl2c3* | 0.017 | 1.762 |  | 19247 | *Gm15645* | 0.015 | 1.462 |
| 4915 | *Prl2c4* | 0.014 | 1.761 |  | 4108 | *Cbr2* | 0.019 | 1.458 |
| 13309 | *Cd101* | 0.049 | 1.760 |  | 20858 | *Gm20735* | 0.048 | 1.454 |
| 12434 | *Snai1* | 0.012 | 1.759 |  | 17120 | *Gata2* | 0.013 | 1.448 |
| 21013 | *Scarna9* | 0.001 | 1.755 |  | 18858 | *Rccd1* | 0.025 | 1.446 |
| 14921 | *Dhrs3* | 0.030 | 1.746 |  | 1974 | *Ric8b* | 0.042 | 1.441 |
| 7715 | *Snord2* | 0.040 | 1.742 |  | 11641 | *Ldlrad3* | 0.024 | 1.440 |
| 10399 | *Ifit1bl2* | 0.012 | 1.730 |  | 7855 | *Ndufb4* | 0.035 | 1.439 |
| 22281 | *2010204K13Rik* | 0.020 | 1.711 |  | 2053 | *Cradd* | 0.021 | 1.438 |
| 13270 | *Pdzk1* | 0.007 | 1.704 |  | 7506 | *Ppl* | 0.012 | 1.438 |
| 13365 | *I830077J02Rik* | 0.045 | 1.684 |  | 12704 | *Slc7a14* | 0.009 | 1.438 |
| 1297 | *Akap12* | 0.011 | 1.647 |  | 4 | *Mrpl15* | 0.037 | 1.437 |
| 4808 | *2810029C07Rik* | 0.002 | 1.634 |  | 18965 | *Tmem126b* | 0.023 | 1.436 |
| 17287 | *Snora7a* | 0.030 | 1.633 |  | 9731 | *Myoz3* | 0.043 | 1.435 |
| 14933 | *Zfp600* | 0.014 | 1.620 |  | 19736 | *Lrrc56* | 0.009 | 1.434 |
| 22158 | *Vill* | 0.006 | 1.616 |  | 21582 | *1600029O15Rik* | 0.033 | 1.432 |
| 19384 | *Pde3b* | 0.031 | 1.615 |  | 4872 | *1700024F13Rik* | 0.029 | 1.427 |
| 12749 | *Bbs12* | 0.044 | 1.613 |  | 9099 | *Zfp119a* | 0.039 | 1.425 |
| 19710 | *Syce1* | 0.035 | 1.610 |  | 4803 | *Exoc3l4* | 0.010 | 1.422 |

| **Gene ID** | **Symbol** | ***p* value** | **Fold Change** |  | **Gene ID** | **Symbol** | ***p* value** | **Fold Change** |
| --- | --- | --- | --- | --- | --- | --- | --- | --- |
| 20756 | *Pdpr* | 0.022 | 1.422 |  | 930 | *4930523C07Rik* | 0.023 | 1.302 |
| 18985 | *Thrsp* | 0.026 | 1.417 |  | 295 | *Sumo1* | 0.022 | 1.301 |
| 16927 | *Vmn1r32* | 0.009 | 1.417 |  | 19241 | *Rrp8* | 0.010 | 1.298 |
| 11811 | *Chac1* | 0.028 | 1.397 |  | 6413 | *R3hcc1* | 0.025 | 1.296 |
| 16937 | *E230016M11Rik* | 0.028 | 1.397 |  | 22736 | *F8* | 0.017 | 1.294 |
| 21840 | *Bcl2a1a* | 0.004 | 1.396 |  | 21739 | *Rab27a* | 0.001 | 1.292 |
| 17711 | *Bicd1* | 0.008 | 1.395 |  | 17903 | *Zfp128* | 0.007 | 1.291 |
| 14628 | *Dcdc2b* | 0.008 | 1.393 |  | 19727 | *Ifitm3* | 0.036 | 1.288 |
| 6800 | *Dscc1* | 0.019 | 1.392 |  | 21810 | *Ttk* | 0.001 | 1.286 |
| 11272 | *Mtx2* | 0.028 | 1.391 |  | 5272 | *Ogn* | 0.040 | 1.282 |
| 1971 | *Tcp11l2* | 0.040 | 1.390 |  | 21502 | *Acat1* | 0.048 | 1.280 |
| 7036 | *Gm10863* | 0.029 | 1.388 |  | 23019 | *Ngfrap1* | 0.003 | 1.274 |
| 5919 | *Selk* | 0.010 | 1.380 |  | 7816 | *Sec22a* | 0.043 | 1.273 |
| 19333 | *Ric3* | 0.000 | 1.379 |  | 11125 | *Gm13498* | 0.008 | 1.272 |
| 16795 | *Igf2bp3* | 0.039 | 1.378 |  | 2018 | *1500026H17Rik* | 0.049 | 1.270 |
| 8760 | *Zbtb12* | 0.010 | 1.371 |  | 15275 | *Insig1* | 0.041 | 1.270 |
| 1089 | *Igsf9* | 0.033 | 1.366 |  | 12408 | *Zfp334* | 0.047 | 1.267 |
| 501 | *Dis3l2* | 0.002 | 1.364 |  | 28 | *Ppp1r42* | 0.023 | 1.266 |
| 10784 | *Il1f9* | 0.001 | 1.356 |  | 12469 | *Cstf1* | 0.002 | 1.264 |
| 134 | *Arid5a* | 0.006 | 1.353 |  | 14241 | *Nfia* | 0.010 | 1.262 |
| 11662 | *Cd59a* | 0.037 | 1.351 |  | 4838 | *Jag2* | 0.031 | 1.261 |
| 21196 | *Cdon* | 0.024 | 1.344 |  | 9958 | *Rce1* | 0.017 | 1.258 |
| 9898 | *Timm21* | 0.016 | 1.344 |  | 21367 | *Pvrl1* | 0.001 | 1.258 |
| 9834 | *Myo5b* | 0.015 | 1.342 |  | 7691 | *Magef1* | 0.044 | 1.256 |
| 7337 | *Asic1* | 0.010 | 1.341 |  | 12237 | *Map1lc3a* | 0.047 | 1.256 |
| 15446 | *Prom1* | 0.010 | 1.341 |  | 5632 | *Ankra2* | 0.009 | 1.252 |
| 16536 | *Tspan12* | 0.010 | 1.341 |  | 11645 | *Slc1a2* | 0.024 | 1.251 |
| 20274 | *Zfp964* | 0.010 | 1.341 |  | 15762 | *Fam175a* | 0.004 | 1.250 |
| 10730 | *Hacd1* | 0.047 | 1.340 |  | 3346 | *Rad51d* | 0.037 | 1.247 |
| 17811 | *Zfp787* | 0.049 | 1.336 |  | 10969 | *Ncs1* | 0.011 | 1.244 |
| 9753 | *Pcyox1l* | 0.002 | 1.333 |  | 476 | *Sp140* | 0.023 | 1.243 |
| 12071 | *Snord17* | 0.049 | 1.332 |  | 6055 | *Ddhd1* | 0.015 | 1.241 |
| 20670 | *Gfod2* | 0.022 | 1.325 |  | 23238 | *Eif2s3y* | 0.016 | 1.241 |
| 3779 | *BC030867* | 0.030 | 1.318 |  | 20915 | *2310022B05Rik* | 0.043 | 1.241 |
| 18496 | *9830147E19Rik* | 0.014 | 1.318 |  | 16143 | *Tpst1* | 0.042 | 1.239 |
| 1989 | *1810014B01Rik* | 0.026 | 1.317 |  | 5348 | *Txndc15* | 0.001 | 1.238 |
| 9535 | *Dnd1* | 0.027 | 1.316 |  | 21010 | *Taf1d* | 0.044 | 1.238 |
| 16403 | *Lnx2* | 0.003 | 1.316 |  | 16892 | *Pyurf* | 0.006 | 1.235 |
| 3854 | *Strada* | 0.024 | 1.316 |  | 3965 | *Sumo2* | 0.045 | 1.234 |
| 16232 | *Pop7* | 0.019 | 1.315 |  | 9702 | *Gramd3* | 0.012 | 1.234 |
| 10069 | *Fkbp2* | 0.028 | 1.310 |  | 13798 | *Manea* | 0.019 | 1.231 |
| 10674 | *Hspa14* | 0.050 | 1.307 |  | 4258 | *Acp1* | 0.004 | 1.228 |
| 8469 | *Hagh* | 0.008 | 1.307 |  | 2061 | *4930459C07Rik* | 0.000 | 1.227 |
| 9973 | *Mrpl11* | 0.006 | 1.306 |  | 3368 | *1700020L24Rik* | 0.000 | 1.227 |
| 5921 | *Il17rb* | 0.000 | 1.303 |  | 6668 | *Golph3* | 0.041 | 1.227 |
| 14927 | *Gm13242* | 0.000 | 1.303 |  | 16368 | *Arpc1b* | 0.032 | 1.226 |

| **Gene ID** | **Symbol** | ***p* value** | **Fold Change** |  | **Gene ID** | **Symbol** | ***p* value** | **Fold Change** |
| --- | --- | --- | --- | --- | --- | --- | --- | --- |
| 14110 | *Whrn* | 0.015 | 1.226 |  | 6433 | *Reep4* | 0.018 | 1.186 |
| 6974 | *Commd5* | 0.012 | 1.225 |  | 15251 | *Prkag2* | 0.030 | 1.186 |
| 20821 | *Klhl36* | 0.029 | 1.224 |  | 10805 | *Fam166a* | 0.039 | 1.184 |
| 3555 | *Snx11* | 0.003 | 1.224 |  | 3027 | *Cyb5d1* | 0.039 | 1.182 |
| 14929 | *Znf41-ps* | 0.049 | 1.224 |  | 12356 | *Wfdc15a* | 0.039 | 1.182 |
| 14547 | *Yrdc* | 0.020 | 1.224 |  | 16401 | *Gtf3a* | 0.036 | 1.182 |
| 12411 | *Trp53rka* | 0.017 | 1.223 |  | 21922 | *Pccb* | 0.038 | 1.180 |
| 3091 | *Med11* | 0.048 | 1.222 |  | 16525 | *Met* | 0.004 | 1.178 |
| 9918 | *Ndufs8* | 0.027 | 1.221 |  | 3496 | *Rsad1* | 0.027 | 1.178 |
| 18309 | *Mrps12* | 0.048 | 1.220 |  | 21085 | *Zglp1* | 0.035 | 1.177 |
| 17659 | *C2cd5* | 0.014 | 1.219 |  | 3816 | *Map3k14* | 0.018 | 1.176 |
| 2312 | *Cdk2* | 0.017 | 1.218 |  | 7309 | *Prkag1* | 0.001 | 1.174 |
| 6907 | *Ly6c2* | 0.015 | 1.216 |  | 16501 | *Glcci1* | 0.023 | 1.173 |
| 152 | *Unc50* | 0.024 | 1.215 |  | 5579 | *Dhfr* | 0.027 | 1.173 |
| 16402 | *Mtif3* | 0.019 | 1.214 |  | 11962 | *Il1b* | 0.009 | 1.172 |
| 1879 | *Abhd17a* | 0.018 | 1.210 |  | 11796 | *Ccdc32* | 0.024 | 1.172 |
| 16794 | *Malsu1* | 0.013 | 1.209 |  | 11974 | *Nop56* | 0.045 | 1.171 |
| 15126 | *Fam133b* | 0.041 | 1.209 |  | 6905 | *Ly6a* | 0.037 | 1.170 |
| 11955 | *Chchd5* | 0.012 | 1.205 |  | 3568 | *Mrpl10* | 0.045 | 1.170 |
| 18899 | *Stard5* | 0.022 | 1.203 |  | 7952 | *Trmt10c* | 0.048 | 1.169 |
| 11696 | *Ccdc34* | 0.029 | 1.202 |  | 348 | *Rpe* | 0.006 | 1.168 |
| 17007 | *Pole4* | 0.032 | 1.201 |  | 11745 | *Emc4* | 0.019 | 1.168 |
| 21928 | *Cep63* | 0.039 | 1.199 |  | 12978 | *Cd5l* | 0.000 | 1.167 |
| 15208 | *Pmpcb* | 0.028 | 1.198 |  | 3489 | *Luc7l3* | 0.020 | 1.167 |
| 13013 | *Pmf1* | 0.034 | 1.197 |  | 17213 | *Ppp4r2* | 0.047 | 1.167 |
| 8462 | *Snora64* | 0.043 | 1.197 |  | 15160 | *Tmem243* | 0.004 | 1.164 |
| 11577 | *Kbtbd4* | 0.046 | 1.195 |  | 21445 | *Rbm7* | 0.027 | 1.163 |
| 6882 | *Ptp4a3* | 0.029 | 1.194 |  | 12302 | *Snhg17* | 0.006 | 1.162 |
| 6654 | *4930556M19Rik* | 0.033 | 1.193 |  | 17426 | *Ing4* | 0.004 | 1.162 |
| 23156 | *Prdx4* | 0.009 | 1.193 |  | 21424 | *Rnf214* | 0.021 | 1.161 |
| 19618 | *Hmx2* | 0.039 | 1.193 |  | 6922 | *Pycrl* | 0.045 | 1.159 |
| 2172 | *Lyz1* | 0.044 | 1.192 |  | 1863 | *Dazap1* | 0.001 | 1.157 |
| 13248 | *Hist2h3b* | 0.026 | 1.192 |  | 11051 | *Ndufa8* | 0.049 | 1.157 |
| 18227 | *B9d2* | 0.008 | 1.192 |  | 10985 | *Uck1* | 0.050 | 1.156 |
| 4918 | *Prl2c5* | 0.042 | 1.190 |  | 20775 | *Terf2ip* | 0.018 | 1.155 |
| 6259 | *Mcpt1* | 0.042 | 1.190 |  | 5343 | *Tmed9* | 0.016 | 1.154 |
| 15065 | *Mmp23* | 0.042 | 1.190 |  | 3745 | *Psme3* | 0.044 | 1.153 |
| 1823 | *Gzmm* | 0.034 | 1.189 |  | 3817 | *Arhgap27* | 0.028 | 1.152 |
| 11109 | *Kynu* | 0.034 | 1.189 |  | 7341 | *Cers5* | 0.002 | 1.152 |
| 18172 | *Tescl* | 0.034 | 1.189 |  | 8009 | *4930547E14Rik* | 0.000 | 1.152 |
| 18412 | *Lgi4* | 0.034 | 1.189 |  | 10919 | *Barhl1* | 0.000 | 1.152 |
| 20113 | *Dctn6* | 0.028 | 1.189 |  | 11956 | *AI847159* | 0.000 | 1.152 |
| 8489 | *Baiap3* | 0.026 | 1.189 |  | 17044 | *1700124L16Rik* | 0.000 | 1.152 |
| 7377 | *Atg101* | 0.044 | 1.187 |  | 18506 | *Siglecg* | 0.000 | 1.152 |
| 13738 | *Ccne2* | 0.000 | 1.187 |  | 21362 | *Tmem136* | 0.000 | 1.152 |
| 21218 | *Chek1* | 0.025 | 1.187 |  | 16023 | *Erp29* | 0.029 | 1.151 |
| 11158 | *Ccdc148* | 0.030 | 1.187 |  | 8617 | *Ccdc167* | 0.039 | 1.150 |

| **Gene ID** | **Symbol** | ***p* value** | **Fold Change** |  | **Gene ID** | **Symbol** | ***p* value** | **Fold Change** |
| --- | --- | --- | --- | --- | --- | --- | --- | --- |
| 22462 | *Ube2a* | 0.049 | 1.149 |  | 19616 | *Acadsb* | 0.022 | 1.108 |
| 4033 | *Tk1* | 0.033 | 1.148 |  | 11031 | *Hspa5* | 0.038 | 1.107 |
| 8526 | *Pigq* | 0.029 | 1.148 |  | 15187 | *Hgf* | 0.021 | 1.106 |
| 781 | *Cyb5r1* | 0.037 | 1.148 |  | 1574 | *Nus1* | 0.004 | 1.105 |
| 3273 | *Rab34* | 0.004 | 1.147 |  | 17762 | *Tmem86b* | 0.020 | 1.104 |
| 5527 | *Zfp72* | 0.001 | 1.144 |  | 6786 | *Eif3h* | 0.027 | 1.100 |
| 21434 | *Zpr1* | 0.017 | 1.144 |  |  |  |  |  |
| 9185 | *Twsg1* | 0.034 | 1.143 |  |  |  |  |  |
| 5482 | *Fastkd3* | 0.030 | 1.141 |  |  |  |  |  |
| 14646 | *Zcchc17* | 0.019 | 1.137 |  |  |  |  |  |
| 17013 | *Loxl3* | 0.043 | 1.137 |  |  |  |  |  |
| 9027 | *Rpl7l1* | 0.018 | 1.137 |  |  |  |  |  |
| 7918 | *Gm4737* | 0.020 | 1.136 |  |  |  |  |  |
| 19515 | *Ino80e* | 0.030 | 1.135 |  |  |  |  |  |
| 17014 | *Htra2* | 0.047 | 1.135 |  |  |  |  |  |
| 12947 | *Plrg1* | 0.033 | 1.134 |  |  |  |  |  |
| 14307 | *Lrrc42* | 0.031 | 1.134 |  |  |  |  |  |
| 14589 | *Smim12* | 0.045 | 1.134 |  |  |  |  |  |
| 2568 | *Mtif2* | 0.012 | 1.133 |  |  |  |  |  |
| 20016 | *Alg11* | 0.046 | 1.133 |  |  |  |  |  |
| 16351 | *Ccz1* | 0.009 | 1.132 |  |  |  |  |  |
| 11800 | *Rmdn3* | 0.017 | 1.130 |  |  |  |  |  |
| 20481 | *Dnase2a* | 0.009 | 1.129 |  |  |  |  |  |
| 20856 | *Mvd* | 0.041 | 1.126 |  |  |  |  |  |
| 11943 | *Bub1* | 0.046 | 1.126 |  |  |  |  |  |
| 9020 | *Mea1* | 0.036 | 1.124 |  |  |  |  |  |
| 5314 | *Cltb* | 0.017 | 1.124 |  |  |  |  |  |
| 4897 | *Wdr37* | 0.004 | 1.124 |  |  |  |  |  |
| 2935 | *Ulk2* | 0.027 | 1.122 |  |  |  |  |  |
| 16055 | *Kdm2b* | 0.011 | 1.122 |  |  |  |  |  |
| 7111 | *Cenpm* | 0.001 | 1.122 |  |  |  |  |  |
| 18263 | *Sertad1* | 0.037 | 1.120 |  |  |  |  |  |
| 4789 | *Hsp90aa1* | 0.012 | 1.120 |  |  |  |  |  |
| 4459 | *Prkch* | 0.030 | 1.120 |  |  |  |  |  |
| 5676 | *Srek1* | 0.029 | 1.120 |  |  |  |  |  |
| 251 | *Hspd1* | 0.049 | 1.120 |  |  |  |  |  |
| 5851 | *Anxa7* | 0.035 | 1.120 |  |  |  |  |  |
| 15128 | *Rbm48* | 0.013 | 1.118 |  |  |  |  |  |
| 4952 | *Aoah* | 0.035 | 1.118 |  |  |  |  |  |
| 20449 | *Asf1b* | 0.040 | 1.115 |  |  |  |  |  |
| 12950 | *Tlr2* | 0.008 | 1.115 |  |  |  |  |  |
| 10865 | *Gpsm1* | 0.036 | 1.113 |  |  |  |  |  |
| 23194 | *Car5b* | 0.005 | 1.113 |  |  |  |  |  |
| 12658 | *Armc1* | 0.047 | 1.112 |  |  |  |  |  |
| 4002 | *Prpsap1* | 0.000 | 1.111 |  |  |  |  |  |
| 14780 | *C1qa* | 0.021 | 1.110 |  |  |  |  |  |
| 7951 | *Pcnp* | 0.025 | 1.110 |  |  |  |  |  |

| Down-regulated gene list | | | | | | | | |
| --- | --- | --- | --- | --- | --- | --- | --- | --- |
|  |  |  |  |  |  |  |  |  |
| **Gene ID** | **Symbol** | ***p* value** | **Fold Change** |  | **Gene ID** | **Symbol** | ***p* value** | **Fold Change** |
| 14864 | *Fblim1* | 0.002 | 0.067 |  | 14865 | *Tmem82* | 0.032 | 0.630 |
| 21934 | *Rab6b* | 0.002 | 0.130 |  | 15699 | *Cxcl9* | 0.018 | 0.630 |
| 14885 | *Pdpn* | 0.001 | 0.136 |  | 13300 | *Spag17* | 0.025 | 0.631 |
| 14801 | *Pink1* | 0.024 | 0.235 |  | 13389 | *Slc16a4* | 0.012 | 0.631 |
| 14943 | *Gm13157* | 0.000 | 0.295 |  | 23062 | *Col4a5* | 0.017 | 0.631 |
| 14949 | *Plod1* | 0.006 | 0.385 |  | 9870 | *Adnp2* | 0.002 | 0.636 |
| 14970 | *Casz1* | 0.009 | 0.419 |  | 20591 | *Zfp319* | 0.042 | 0.642 |
| 14945 | *Zfp933* | 0.007 | 0.432 |  | 138 | *Cnnm4* | 0.017 | 0.643 |
| 20794 | *1700030J22Rik* | 0.010 | 0.495 |  | 18249 | *Cyp2t4* | 0.017 | 0.646 |
| 14582 | *AU040320* | 0.010 | 0.514 |  | 5563 | *Edil3* | 0.007 | 0.647 |
| 20018 | *Nek3* | 0.006 | 0.519 |  | 5456 | *Zfp458* | 0.024 | 0.648 |
| 20483 | *Gm38426* | 0.044 | 0.533 |  | 7190 | *Alg12* | 0.016 | 0.649 |
| 444 | *Utp14b* | 0.034 | 0.537 |  | 16156 | *Gtf2ird2* | 0.022 | 0.649 |
| 7181 | *Celsr1* | 0.020 | 0.538 |  | 1352 | *Adgrg6* | 0.027 | 0.652 |
| 3425 | *Ypel2* | 0.048 | 0.540 |  | 5084 | *Lrrc16a* | 0.035 | 0.653 |
| 1247 | *Vash2* | 0.007 | 0.544 |  | 10393 | *Ch25h* | 0.017 | 0.656 |
| 1539 | *Bend3* | 0.022 | 0.553 |  | 19768 | *Brsk2* | 0.004 | 0.657 |
| 7571 | *Myh11* | 0.003 | 0.556 |  | 14923 | *Tnfrsf1b* | 0.020 | 0.661 |
| 12162 | *Trib3* | 0.010 | 0.556 |  | 8319 | *Has1* | 0.036 | 0.661 |
| 14676 | *Phactr4* | 0.030 | 0.557 |  | 9945 | *Clcf1* | 0.046 | 0.662 |
| 8409 | *Flywch1* | 0.024 | 0.565 |  | 3424 | *Dhx40* | 0.025 | 0.663 |
| 9399 | *Kctd1* | 0.006 | 0.567 |  | 16819 | *Hoxaas2* | 0.003 | 0.663 |
| 5606 | *Zbed3* | 0.025 | 0.572 |  | 17393 | *Cd163* | 0.003 | 0.663 |
| 22542 | *Zfp280c* | 0.001 | 0.573 |  | 10837 | *Mamdc4* | 0.034 | 0.665 |
| 4160 | *Ubxn2a* | 0.013 | 0.573 |  | 1499 | *E130307A14Rik* | 0.021 | 0.667 |
| 13318 | *Vangl1* | 0.008 | 0.577 |  | 7871 | *Tmem39a* | 0.049 | 0.669 |
| 23189 | *S100g* | 0.028 | 0.583 |  | 22089 | *Elp6* | 0.029 | 0.670 |
| 10044 | *Batf2* | 0.028 | 0.586 |  | 3121 | *Nlrp1a* | 0.043 | 0.674 |
| 5206 | *Gcnt2* | 0.041 | 0.588 |  | 12127 | *3300002I08Rik* | 0.048 | 0.676 |
| 156 | *Tsga10* | 0.015 | 0.590 |  | 16447 | *Stard13* | 0.001 | 0.680 |
| 14758 | *Cnr2* | 0.033 | 0.595 |  | 22805 | *Zxdb* | 0.042 | 0.682 |
| 21543 | *Cspg4* | 0.018 | 0.600 |  | 4312 | *Dock4* | 0.041 | 0.684 |
| 1342 | *Plagl1* | 0.030 | 0.600 |  | 6617 | *Card6* | 0.047 | 0.685 |
| 11925 | *1810024B03Rik* | 0.003 | 0.607 |  | 13254 | *Gm15441* | 0.047 | 0.687 |
| 5855 | *Usp54* | 0.020 | 0.610 |  | 6236 | *Tssk4* | 0.014 | 0.687 |
| 522 | *Ugt1a10* | 0.023 | 0.610 |  | 14782 | *Zbtb40* | 0.050 | 0.688 |
| 20454 | *Palm3* | 0.035 | 0.614 |  | 12841 | *Gpr171* | 0.010 | 0.688 |
| 15601 | *Hopx* | 0.005 | 0.615 |  | 700 | *Zranb3* | 0.009 | 0.689 |
| 10720 | *Itga8* | 0.012 | 0.618 |  | 11681 | *Pax6* | 0.049 | 0.690 |
| 16020 | *Gm15800* | 0.046 | 0.619 |  | 22379 | *Gpr82* | 0.010 | 0.690 |
| 9974 | *Npas4* | 0.000 | 0.619 |  | 20119 | *Tnks* | 0.038 | 0.693 |
| 44 | *Eya1* | 0.027 | 0.622 |  | 14154 | *Ttc39b* | 0.028 | 0.695 |
| 18654 | *Lmtk3* | 0.044 | 0.622 |  | 834 | *Zbtb41* | 0.004 | 0.695 |
| 22893 | *Zdhhc15* | 0.046 | 0.625 |  | 640 | *Bcl2* | 0.025 | 0.695 |

| **Gene ID** | **Symbol** | ***p* value** | **Fold Change** |  | **Gene ID** | **Symbol** | ***p* value** | **Fold Change** |
| --- | --- | --- | --- | --- | --- | --- | --- | --- |
| 2747 | *D930048N14Rik* | 0.004 | 0.697 |  | 17503 | *Clec9a* | 0.025 | 0.753 |
| 7146 | *Ttll12* | 0.021 | 0.702 |  | 14530 | *Macf1* | 0.037 | 0.753 |
| 5869 | *Kat6b* | 0.047 | 0.702 |  | 14288 | *Ppap2b* | 0.010 | 0.753 |
| 11140 | *Fmnl2* | 0.028 | 0.705 |  | 13488 | *Gclm* | 0.024 | 0.754 |
| 5677 | *Erbb2ip* | 0.047 | 0.711 |  | 18414 | *Hpn* | 0.003 | 0.755 |
| 11825 | *Mapkbp1* | 0.032 | 0.714 |  | 16374 | *Zkscan14* | 0.049 | 0.755 |
| 4639 | *5430427M07Rik* | 0.034 | 0.714 |  | 3486 | *Spag9* | 0.022 | 0.756 |
| 9703 | *Aldh7a1* | 0.049 | 0.715 |  | 9140 | *Dus3l* | 0.030 | 0.757 |
| 9887 | *Zfp516* | 0.016 | 0.715 |  | 15383 | *Rgs12* | 0.037 | 0.758 |
| 9874 | *Hsbp1l1* | 0.001 | 0.716 |  | 15767 | *Arhgap24* | 0.018 | 0.759 |
| 1025 | *3110045C21Rik* | 0.026 | 0.717 |  | 15895 | *Adrbk2* | 0.037 | 0.759 |
| 10988 | *Dnm1* | 0.029 | 0.717 |  | 4937 | *Cdk13* | 0.012 | 0.759 |
| 11864 | *Serinc4* | 0.005 | 0.720 |  | 12319 | *Zhx3* | 0.013 | 0.761 |
| 7652 | *Hira* | 0.025 | 0.723 |  | 6344 | *Fdft1* | 0.040 | 0.762 |
| 3725 | *Ptrf* | 0.013 | 0.723 |  | 15465 | *5730480H06Rik* | 0.017 | 0.762 |
| 4960 | *Zscan12* | 0.005 | 0.725 |  | 16663 | *4930599N23Rik* | 0.023 | 0.762 |
| 205 | *Uxs1* | 0.019 | 0.725 |  | 6360 | *Msra* | 0.037 | 0.762 |
| 10087 | *Snord118* | 0.025 | 0.725 |  | 5072 | *Hist1h4b* | 0.001 | 0.763 |
| 4921 | *Lyst* | 0.004 | 0.726 |  | 18875 | *Pde8a* | 0.035 | 0.764 |
| 7054 | *Gm16576* | 0.003 | 0.727 |  | 16850 | *Plekha8* | 0.013 | 0.765 |
| 3303 | *Nf1* | 0.035 | 0.729 |  | 16512 | *Tmem168* | 0.014 | 0.766 |
| 1024 | *Hsd17b7* | 0.014 | 0.732 |  | 13302 | *Gdap2* | 0.037 | 0.767 |
| 94 | *Fam135a* | 0.012 | 0.732 |  | 6971 | *Arhgap39* | 0.032 | 0.767 |
| 7515 | *Mettl22* | 0.024 | 0.732 |  | 2051 | *Plxnc1* | 0.017 | 0.767 |
| 4583 | *Mlh3* | 0.009 | 0.732 |  | 6921 | *Tigd5* | 0.033 | 0.768 |
| 15911 | *Ssh1* | 0.043 | 0.734 |  | 6362 | *Hmbox1* | 0.043 | 0.769 |
| 637 | *Zcchc2* | 0.010 | 0.735 |  | 3807 | *Dcakd* | 0.037 | 0.770 |
| 12501 | *Edn3* | 0.021 | 0.736 |  | 6627 | *Rictor* | 0.020 | 0.770 |
| 14298 | *Fam151a* | 0.034 | 0.738 |  | 17350 | *Pex26* | 0.027 | 0.771 |
| 16858 | *Fam188b* | 0.034 | 0.738 |  | 889 | *Rnasel* | 0.044 | 0.772 |
| 10154 | *Tmem138* | 0.022 | 0.738 |  | 21889 | *Nmnat3* | 0.042 | 0.773 |
| 392 | *Vil1* | 0.012 | 0.738 |  | 9398 | *Taf4b* | 0.010 | 0.774 |
| 10983 | *Prrc2b* | 0.035 | 0.738 |  | 7059 | *D730005E14Rik* | 0.044 | 0.774 |
| 20387 | *Slc35e1* | 0.001 | 0.741 |  | 17033 | *Tet3* | 0.011 | 0.775 |
| 5150 | *Serpinb9* | 0.001 | 0.741 |  | 9519 | *Igip* | 0.009 | 0.777 |
| 14749 | *Nipal3* | 0.037 | 0.742 |  | 12316 | *Mafb* | 0.006 | 0.777 |
| 4670 | *Ccdc88c* | 0.012 | 0.742 |  | 6388 | *Pnma2* | 0.038 | 0.777 |
| 2585 | *Cpeb4* | 0.019 | 0.745 |  | 9250 | *Eif2ak2* | 0.025 | 0.778 |
| 15194 | *Magi2* | 0.016 | 0.746 |  | 14877 | *Efhd2* | 0.031 | 0.779 |
| 19043 | *Fam168a* | 0.033 | 0.746 |  | 18352 | *Zfp260* | 0.014 | 0.779 |
| 11003 | *Fpgs* | 0.046 | 0.746 |  | 14888 | *Pramef8* | 0.001 | 0.780 |
| 6275 | *Parp4* | 0.047 | 0.748 |  | 7136 | *Rnu12* | 0.047 | 0.781 |
| 14632 | *Kpna6* | 0.032 | 0.749 |  | 15025 | *Nphp4* | 0.047 | 0.781 |
| 7615 | *Slc7a4* | 0.019 | 0.749 |  | 3474 | *Mmd* | 0.021 | 0.781 |
| 22889 | *Rlim* | 0.035 | 0.750 |  | 7593 | *Top3b* | 0.017 | 0.781 |
| 1628 | *Adamts14* | 0.025 | 0.753 |  | 6408 | *Slc25a37* | 0.041 | 0.781 |
| 5689 | *4933425L06Rik* | 0.025 | 0.753 |  | 17687 | *Ppfibp1* | 0.029 | 0.781 |

| **Gene ID** | **Symbol** | ***p* value** | **Fold Change** |  | **Gene ID** | **Symbol** | ***p* value** | **Fold Change** |
| --- | --- | --- | --- | --- | --- | --- | --- | --- |
| 5978 | *Arhgap22* | 0.017 | 0.782 |  | 4297 | *Ankmy2* | 0.027 | 0.813 |
| 17506 | *Olr1* | 0.014 | 0.784 |  | 15808 | *Btbd8* | 0.032 | 0.813 |
| 809 | *Tmem9* | 0.007 | 0.784 |  | 20937 | *Irf2bp2* | 0.011 | 0.815 |
| 18633 | *Nucb1* | 0.037 | 0.785 |  | 16289 | *D830046C22Rik* | 0.043 | 0.815 |
| 18345 | *Zfp27* | 0.002 | 0.785 |  | 5420 | *2010111I01Rik* | 0.021 | 0.817 |
| 5135 | *Dusp22* | 0.000 | 0.786 |  | 6995 | *Myh9* | 0.026 | 0.817 |
| 3108 | *Camta2* | 0.001 | 0.787 |  | 19585 | *9130023H24Rik* | 0.024 | 0.817 |
| 21098 | *Slc44a2* | 0.050 | 0.788 |  | 1019 | *Pbx1* | 0.038 | 0.817 |
| 23186 | *Txlng* | 0.018 | 0.788 |  | 14127 | *Aldoart1* | 0.037 | 0.817 |
| 19555 | *Phkg2* | 0.049 | 0.789 |  | 8125 | *Ifnar1* | 0.006 | 0.817 |
| 7693 | *2510009E07Rik* | 0.004 | 0.789 |  | 17209 | *Rybp* | 0.014 | 0.819 |
| 3628 | *Wipf2* | 0.038 | 0.792 |  | 15759 | *Hpse* | 0.012 | 0.820 |
| 299 | *Bmpr2* | 0.046 | 0.794 |  | 19566 | *Hsd3b7* | 0.030 | 0.821 |
| 18865 | *Crtc3* | 0.030 | 0.795 |  | 3860 | *Tcam1* | 0.003 | 0.821 |
| 8435 | *Abca3* | 0.018 | 0.795 |  | 9170 | *Efna5* | 0.003 | 0.821 |
| 18452 | *Gpatch1* | 0.023 | 0.795 |  | 22978 | *Drp2* | 0.003 | 0.821 |
| 14692 | *Wasf2* | 0.017 | 0.795 |  | 246 | *Gtf3c3* | 0.046 | 0.821 |
| 11653 | *Cat* | 0.026 | 0.796 |  | 3564 | *Sp2* | 0.045 | 0.821 |
| 9515 | *Psd2* | 0.036 | 0.796 |  | 11869 | *Frmd5* | 0.049 | 0.822 |
| 9787 | *Ccbe1* | 0.036 | 0.796 |  | 16428 | *Uspl1* | 0.013 | 0.822 |
| 17649 | *Spx* | 0.036 | 0.796 |  | 20526 | *Adcy7* | 0.004 | 0.824 |
| 9351 | *Svil* | 0.011 | 0.797 |  | 3834 | *Cdc27* | 0.002 | 0.825 |
| 15790 | *Gbp8* | 0.019 | 0.797 |  | 7894 | *Spice1* | 0.029 | 0.826 |
| 18050 | *Apoe* | 0.032 | 0.798 |  | 14745 | *Clic4* | 0.043 | 0.827 |
| 4041 | *Cyth1* | 0.010 | 0.798 |  | 17230 | *Bhlhe40* | 0.004 | 0.827 |
| 1327 | *Samd5* | 0.008 | 0.799 |  | 19597 | *Wdr11* | 0.046 | 0.827 |
| 6437 | *Dmtn* | 0.008 | 0.799 |  | 10163 | *Cd5* | 0.039 | 0.829 |
| 19556 | *Gm166* | 0.022 | 0.799 |  | 14573 | *Tekt2* | 0.041 | 0.829 |
| 3145 | *Cyb5d2* | 0.009 | 0.799 |  | 8739 | *Notch4* | 0.025 | 0.829 |
| 7310 | *Kmt2d* | 0.033 | 0.803 |  | 9392 | *Impact* | 0.024 | 0.830 |
| 13312 | *Igsf3* | 0.011 | 0.804 |  | 6161 | *Zfp219* | 0.023 | 0.830 |
| 1575 | *Zfa-ps* | 0.028 | 0.804 |  | 11159 | *Pkp4* | 0.017 | 0.830 |
| 12200 | *Asxl1* | 0.007 | 0.805 |  | 15560 | *Slain2* | 0.032 | 0.830 |
| 2123 | *E2f7* | 0.000 | 0.805 |  | 16032 | *Atxn2* | 0.018 | 0.831 |
| 4479 | *Hspa2* | 0.017 | 0.806 |  | 3219 | *Slc43a2* | 0.019 | 0.831 |
| 20613 | *Tk2* | 0.009 | 0.806 |  | 14228 | *Jun* | 0.019 | 0.832 |
| 16442 | *Brca2* | 0.021 | 0.807 |  | 16061 | *Setd1b* | 0.014 | 0.833 |
| 3576 | *Socs7* | 0.029 | 0.808 |  | 14975 | *Pgd* | 0.035 | 0.833 |
| 8231 | *Rsph3a* | 0.017 | 0.808 |  | 19573 | *Bckdk* | 0.021 | 0.833 |
| 14730 | *Pafah2* | 0.020 | 0.809 |  | 13922 | *Rgp1* | 0.042 | 0.834 |
| 21673 | *Rbpms2* | 0.017 | 0.809 |  | 13641 | *Spata1* | 0.014 | 0.835 |
| 6738 | *Ncald* | 0.042 | 0.810 |  | 19627 | *Fam53b* | 0.040 | 0.835 |
| 3920 | *Cog1* | 0.040 | 0.812 |  | 22305 | *Hdac6* | 0.004 | 0.835 |
| 3426 | *Gdpd1* | 0.039 | 0.812 |  | 17704 | *Ipo8* | 0.031 | 0.836 |
| 10982 | *Ppapdc3* | 0.040 | 0.812 |  | 4961 | *Zkscan3* | 0.049 | 0.837 |
| 15928 | *Git2* | 0.006 | 0.812 |  | 3751 | *Rundc1* | 0.049 | 0.837 |
| 4874 | *Fam208b* | 0.000 | 0.813 |  | 13512 | *Camk2d* | 0.025 | 0.838 |

| **Gene ID** | **Symbol** | ***p* value** | **Fold Change** |  | **Gene ID** | **Symbol** | ***p* value** | **Fold Change** |
| --- | --- | --- | --- | --- | --- | --- | --- | --- |
| 9005 | *Dlk2* | 0.042 | 0.839 |  | 390 | *Slc11a1* | 0.035 | 0.860 |
| 3573 | *Npepps* | 0.047 | 0.839 |  | 4439 | *Daam1* | 0.018 | 0.860 |
| 6725 | *Vps13b* | 0.044 | 0.839 |  | 9380 | *Rbbp8* | 0.041 | 0.861 |
| 15657 | *Rufy3* | 0.000 | 0.840 |  | 4551 | *Acot4* | 0.041 | 0.861 |
| 7544 | *Zc3h7a* | 0.013 | 0.840 |  | 6312 | *Mtmr6* | 0.040 | 0.862 |
| 17339 | *Kdm5a* | 0.046 | 0.840 |  | 1255 | *Atf3* | 0.044 | 0.862 |
| 1958 | *Txnrd1* | 0.004 | 0.841 |  | 21597 | *Arih1* | 0.036 | 0.864 |
| 10102 | *Slc3a2* | 0.041 | 0.842 |  | 9064 | *Nfya* | 0.005 | 0.864 |
| 8436 | *D330041H03Rik* | 0.043 | 0.843 |  | 21788 | *Slc17a5* | 0.032 | 0.864 |
| 4764 | *Yy1* | 0.016 | 0.844 |  | 4460 | *Hif1a* | 0.001 | 0.864 |
| 8066 | *Rwdd2b* | 0.047 | 0.844 |  | 23114 | *Huwe1* | 0.011 | 0.864 |
| 12007 | *Ap5s1* | 0.025 | 0.844 |  | 14849 | *Necap2* | 0.018 | 0.865 |
| 19593 | *Inpp5f* | 0.040 | 0.845 |  | 7555 | *Ercc4* | 0.012 | 0.865 |
| 2158 | *Cnot2* | 0.006 | 0.845 |  | 927 | *Rfwd2* | 0.019 | 0.866 |
| 8316 | *Lnpep* | 0.027 | 0.845 |  | 18486 | *AW146154* | 0.009 | 0.866 |
| 18626 | *Lhb* | 0.018 | 0.846 |  | 4998 | *Hist1h2bk* | 0.049 | 0.866 |
| 3263 | *Flot2* | 0.008 | 0.846 |  | 14324 | *Zyg11b* | 0.006 | 0.866 |
| 9386 | *Npc1* | 0.029 | 0.846 |  | 1170 | *Psen2* | 0.002 | 0.867 |
| 902 | *Xpr1* | 0.036 | 0.847 |  | 21099 | *Ilf3* | 0.031 | 0.868 |
| 1590 | *Hsf2* | 0.044 | 0.848 |  | 15509 | *Fam114a1* | 0.002 | 0.870 |
| 1496 | *Wisp3* | 0.021 | 0.848 |  | 6957 | *Cpsf1* | 0.026 | 0.871 |
| 7906 | *Btla* | 0.021 | 0.848 |  | 5958 | *Ankrd28* | 0.049 | 0.871 |
| 13908 | *Atp8b5* | 0.021 | 0.848 |  | 17955 | *C5ar2* | 0.002 | 0.872 |
| 16036 | *Myl2* | 0.021 | 0.848 |  | 14711 | *Rps6ka1* | 0.008 | 0.873 |
| 19369 | *Micalcl* | 0.021 | 0.848 |  | 5429 | *Habp4* | 0.007 | 0.873 |
| 9455 | *Ammecr1l* | 0.018 | 0.848 |  | 3637 | *Krt222* | 0.003 | 0.873 |
| 6862 | *Sla* | 0.028 | 0.849 |  | 4632 | *Gm7104* | 0.003 | 0.873 |
| 4602 | *Irf2bpl* | 0.014 | 0.849 |  | 10000 | *Ctsw* | 0.003 | 0.873 |
| 5763 | *Zfp131* | 0.048 | 0.852 |  | 10707 | *Itih2* | 0.003 | 0.873 |
| 22024 | *Mst1* | 0.044 | 0.852 |  | 13311 | *Cd2* | 0.003 | 0.873 |
| 4003 | *Sphk1* | 0.046 | 0.853 |  | 13398 | *Eps8l3* | 0.003 | 0.873 |
| 5659 | *Marveld2* | 0.046 | 0.853 |  | 17485 | *Klrb1c* | 0.003 | 0.873 |
| 14528 | *Bmp8a* | 0.046 | 0.853 |  | 18557 | *1700008O03Rik* | 0.003 | 0.873 |
| 1965 | *Appl2* | 0.018 | 0.853 |  | 2144 | *Tbc1d15* | 0.023 | 0.873 |
| 4588 | *Fos* | 0.015 | 0.853 |  | 2716 | *Sqstm1* | 0.008 | 0.874 |
| 17198 | *Tmf1* | 0.007 | 0.853 |  | 4326 | *G2e3* | 0.042 | 0.874 |
| 21234 | *Siae* | 0.023 | 0.854 |  | 13575 | *Ppp3ca* | 0.035 | 0.874 |
| 16944 | *Rpia* | 0.039 | 0.854 |  | 2937 | *Specc1* | 0.041 | 0.875 |
| 10522 | *Fam178a* | 0.044 | 0.855 |  | 2175 | *Mdm2* | 0.014 | 0.875 |
| 14277 | *Mier1* | 0.012 | 0.855 |  | 1241 | *Cenpf* | 0.043 | 0.876 |
| 16033 | *Sh2b3* | 0.025 | 0.855 |  | 9679 | *Dmxl1* | 0.029 | 0.876 |
| 10138 | *Fth1* | 0.047 | 0.857 |  | 2921 | *Usp22* | 0.007 | 0.876 |
| 5371 | *Gkap1* | 0.037 | 0.858 |  | 22203 | *Zfp651* | 0.050 | 0.878 |
| 7260 | *Arid2* | 0.023 | 0.858 |  | 15405 | *D5Ertd579e* | 0.018 | 0.878 |
| 7604 | *Hic2* | 0.006 | 0.859 |  | 11139 | *Stam2* | 0.046 | 0.878 |
| 17282 | *Mkrn2* | 0.023 | 0.859 |  | 5390 | *Zcchc6* | 0.028 | 0.878 |
| 11669 | *Pin1rt1* | 0.017 | 0.859 |  | 2146 | *Tmem19* | 0.042 | 0.879 |

| **Gene ID** | **Symbol** | ***p* value** | **Fold Change** |
| --- | --- | --- | --- |
| 6383 | *Trim35* | 0.045 | 0.880 |
| 8058 | *App* | 0.024 | 0.881 |
| 6535 | *Fbxl3* | 0.010 | 0.882 |
| 15858 | *Ankle2* | 0.026 | 0.882 |
| 20109 | *Gsr* | 0.037 | 0.882 |
| 21556 | *1700017B05Rik* | 0.003 | 0.883 |
| 2656 | *Cyfip2* | 0.042 | 0.883 |
| 8541 | *Dusp1* | 0.029 | 0.883 |
| 7152 | *Pnpla3* | 0.006 | 0.883 |
| 16087 | *2810006K23Rik* | 0.024 | 0.886 |
| 7573 | *Abcc1* | 0.042 | 0.887 |
| 6929 | *Scrib* | 0.038 | 0.887 |
| 3302 | *Wsb1* | 0.002 | 0.887 |
| 21192 | *Srpr* | 0.024 | 0.887 |
| 7365 | *Galnt6* | 0.009 | 0.887 |
| 2465 | *Ogdh* | 0.039 | 0.888 |
| 8556 | *Itpr3* | 0.038 | 0.889 |
| 894 | *Glul* | 0.023 | 0.890 |
| 6046 | *Ptger2* | 0.016 | 0.890 |
| 1591 | *Serinc1* | 0.039 | 0.891 |
| 961 | *Prrc2c* | 0.029 | 0.891 |
| 15853 | *Gtpbp6* | 0.028 | 0.893 |
| 1152 | *Akt3* | 0.046 | 0.893 |
| 12719 | *Ttc14* | 0.037 | 0.894 |
| 15852 | *Plcxd1* | 0.029 | 0.895 |
| 20112 | *Rbpms* | 0.017 | 0.895 |
| 2238 | *Os9* | 0.002 | 0.897 |
| 5694 | *Kif2a* | 0.032 | 0.897 |
| 10419 | *Kif11* | 0.049 | 0.899 |
| 5829 | *Top2b* | 0.022 | 0.899 |
| 6076 | *Ktn1* | 0.048 | 0.900 |
| 9373 | *Esco1* | 0.047 | 0.900 |
| 17329 | *Adipor2* | 0.015 | 0.903 |
| 16028 | *Aldh2* | 0.030 | 0.903 |
| 12775 | *Sclt1* | 0.042 | 0.905 |
| 9418 | *B4galt6* | 0.040 | 0.905 |
| 20404 | *Arhgap10* | 0.003 | 0.905 |
| 13773 | *Wwp1* | 0.023 | 0.908 |
| 7761 | *Acap2* | 0.015 | 0.908 |
| 5613 | *Iqgap2* | 0.022 | 0.908 |
